# The Arabidopsis *miR472-RDR6* Silencing Pathway Modulates PAMP- and Effector-Triggered Immunity through the Post-transcriptional Control of Disease Resistance Genes

**DOI:** 10.1371/journal.ppat.1003883

**Published:** 2014-01-16

**Authors:** Martine Boccara, Alexis Sarazin, Odon Thiébeauld, Florence Jay, Olivier Voinnet, Lionel Navarro, Vincent Colot

**Affiliations:** 1 Institut de Biologie de l'Ecole Normale Supérieure (IBENS), Centre National de la Recherche Scientifique UMR8197, Institut National de la Santé et de la Recherche Médicale U1024, Paris, France; 2 Université Pierre et Marie Curie, Paris, France; 3 ETH Zurich, Department of Biology, Chair of RNA biology, Zurich, Switzerland; Chinese Academy of Sciences, China

## Abstract

RNA-DEPENDENT RNA POLYMERASE 6 (RDR6) is a key RNA silencing factor initially characterized in transgene silencing and virus resistance. This enzyme also contributes to the biosynthesis of endogenous short interfering RNAs (siRNAs) from non-coding RNAs, transposable elements and protein-coding transcripts. One class of protein-coding transcripts that have recently emerged as major sources of *RDR6*-dependent siRNAs are nucleotide-binding leucine-rich repeat (NB-LRR) proteins, a family of immune-receptors that perceive specific pathogen effector proteins and mount Effector-Triggered Immunity (ETI). Nevertheless, the dynamic post-transcriptional control of NB-LRR transcripts during the plant immune response and the functional relevance of NB-LRRs in signaling events triggered by Pathogen-Associated Molecular Patterns (PAMPs) remain elusive. Here, we show that PTI is constitutive and sensitized in the Arabidopsis *rdr6* loss-of-function mutant, implicating *RDR6* as a novel negative regulator of PTI. Accordingly, *rdr6* mutant exhibits enhanced basal resistance towards a virulent *Pseudomonas syringae* strain. We further provide evidence that dozens of CC-NB-LRRs (CNLs), including the functionally characterized *RPS5* gene, are post-transcriptionally controlled by *RDR6* both constitutively and during PTI. These CNL transcripts are also regulated by the Arabidopsis microRNA *miR472* and knock-down of this miRNA recapitulates the PTI and basal resistance phenotypes observed in the *rdr6* mutant background. Furthermore, both *miR472* and *rdr6* mutants were more resistant to *Pto* DC3000 expressing AvrPphB, a bacterial effector recognized by the disease resistance protein RPS5, whereas transgenic plants overexpressing *miR472* were more susceptible to this bacterial strain. Finally, we show that the enhanced basal and *RPS5*-mediated resistance phenotypes observed in the *rdr6* mutant are dependent on the proper chaperoning of NB-LRR proteins, and might therefore be due to the enhanced accumulation of CNL proteins whose cognate mRNAs are no longer controlled by *RDR6*-dependent siRNAs. Altogether, this study supports a model whereby the miR472- and RDR6-mediated silencing pathway represents a key regulatory checkpoint modulating both PTI and ETI responses through the post-transcriptional control of disease resistance genes.

## Author Summary

Virus resistance relies in some plant-viral interactions on the *RNA-DEPENDANT RNA POLYMERASE 6* (*RDR6*), a major actor of RNA silencing that acts at the post-transcriptional level. Here, we demonstrate that *RDR6* also plays a role in basal defense and race-specific resistance. *RDR6* and the microRNA *miR472*, which targets the mRNAs of disease resistance genes of coiled-coil nucleotide-binding leucine-rich-repeats family (e.g. *RPS5*), act in cooperation to control post-transcriptionally these immune receptors. Induction of these resistance genes is primed in *rdr6-* and *miR472*-elicited mutants and this effect is associated with an enhanced basal and race-specific immunity in these backgrounds.

## Introduction

To defend themselves against pathogens, plants have evolved potent inducible immune responses. The first line of active defense relies on the recognition of common features of microbial pathogens, such as flagellin (the major protein of bacterial flagellum), lipopolysaccharides, glycoproteins and chitin [1]. These microbial determinants are referred to as Pathogen- or Microbe- Associated Molecular Patterns (PAMPs/MAMPs) and are sensed by host-encoded Pattern-Recognition Receptors (PRRs) or surface receptors, which encode transmembrane receptor-like kinases. Upon PAMP detection, PRRs trigger a series of immune responses including, for instance, MAPK (mitogen-activated protein kinase) activation, reactive oxygen species (ROS) production, differential expression of genes, callose (β-1->3 glucose polymer) deposition and stomatal closure, which ultimately leads to basal immunity or PAMP-Triggered Immunity (PTI) [2]–[5]. To enable disease, pathogens produce a large array of divergent virulent determinants known as pathogen effectors that suppress different steps of PTI, resulting in disease susceptibility [6], [7]. As a counter-counter defense strategy, plants have evolved a repertoire of immune receptors, called disease resistance (R) proteins that can sense effector proteins and establish effector-triggered-immunity (ETI) [1]. The largest class of R proteins is composed of intracellular receptors that share structural homologies with mammalian innate immune receptors, such as NUCLEOTIDE-BINDING OLIGOMERIZATION DOMAIN-CONTAINING PROTEIN 1 (NOD1) and NOD2, which perceive bacterial PAMPs [8]. Plant NOD-like receptors (NLRs) are composed of nucleotide-binding (NB) and leucine-rich repeat (LRR) domains. They additionally contain an N-terminal domain that is composed of either a Toll/interleukin1 receptor (TIR) or a coiled-coil (CC) module, and are thus referred to as TNLs or CNLs, respectively [9]. These R proteins can directly sense pathogen effectors [1], however, in most cases they recognize indirectly these virulent determinants by detecting their effects on plant target proteins called ‘guardees’ [10]. Upon pathogen effector recognition, R proteins trigger a series of immune responses that significantly overlap with PTI responses, albeit with a stronger amplitude, and often result in a form of programmed cell death known as the hypersensitive response (HR) [1]. Importantly, constitutive expression or activation of R proteins often leads to constitutive cell death as well as severe developmental defects in the absence of pathogen [11]–[16], indicating that *R* genes and their products must be under tight negative control in unchallenged conditions. Consistent with this idea, transcriptional regulation, RNA processing, protein modifications, protein stability, and nucleocytoplasmic trafficking were shown to play a critical role in controlling R-mediated autoimmune responses [17].

More recently, RNA silencing has also emerged as a key regulatory mechanism that negatively regulates *R* gene expression [18]–[24]. RNA silencing is an ancestral gene regulatory mechanism that controls gene expression at the transcriptional (TGS, Transcriptional Gene Silencing) and post-transcriptional (PTGS, Post-transcriptional Gene Silencing) levels. The core mechanism of RNA silencing starts with the production of double stranded RNAs (dsRNAs) that are processed by RNase-III enzymes DICERs into 20–24 nt small RNA duplexes. One selected strand is subsequently incorporated into an RNA-induced silencing complex (RISC) containing an argonaute (AGO) protein, and guides these complexes onto sequence complementary RNA/DNA targets. The plant model *Arabidopsis thaliana* encodes 4 DICER-like proteins and 10 AGOs. DCL1 processes miRNA precursors into mature microRNAs that are mostly incorporated into the AGO1-RISC that guides mRNA degradation and/or translation inhibition of sequence complementary mRNA targets. DCL2, DCL3 and DCL4 are involved in the biogenesis of short interfering RNAs (siRNAs) from extensive dsRNAs produced from read through, convergent or overlapping transcription, endogenous hairpins as well as some miRNA precursors [25], [26]. As an example, overlapping sense and antisense transcripts that are produced at a functionally relevant disease resistance gene cluster, were found to be processed into siRNAs, leading to the down-regulation of several disease resistance gene transcripts within this cluster [18]. In addition, a large proportion of dsRNAs are produced by RNA-dependent RNA polymerases (RDRs) that convert single stranded RNAs into dsRNAs. RDR6, which is one out of six Arabidopsis RDRs, produces dsRNAs from viral and transgene transcripts as well as some endogenous transcripts [27]. These dsRNAs are processed in part by DCL4 into 21 nt siRNAs that direct PTGS of endogenous mRNA targets or exogenous RNAs derived from sense-transgenes or viral RNAs [28]–[31]. In addition to the biogenesis of primary siRNAs, plants have evolved the production of secondary siRNAs as a feed-forward amplification of silencing signals. These siRNAs are produced by the combined action of primary siRNA/miRNA-directed transcript cleavage and the activity of RDRs that use the target transcripts as template to generate dsRNAs [32]. In plants, the best-characterized endogenous secondary siRNAs are termed *trans*-acting siRNAs (tasiRNAs) [33], [34]. The biogenesis of these small RNA molecules is initiated by 22 nt long miRNAs that direct AGO1-mediated cleavage of a non-coding TAS primary transcript [35], [36]. One of the cleavage products is then converted by RDR6 into dsRNAs, which are processed by DCL4 into 21-nt phased siRNA duplexes. These secondary siRNAs guide an AGO protein to silence sequence complementary mRNA targets *in trans*. Importantly, this phenomenon is not restricted to non-coding transcripts but also targets protein-coding transcripts and both TNLs and CNLs have emerged as major targets of this silencing pathway [20], [21], [22]. For example, two 22 nt long miRNAs that initiate the production of RDR6-dependent secondary siRNAs, were found to directly control the tobacco disease resistance gene *N*, which recognizes the C-terminal helicase domain of the *Tobacco Mosaic Virus* (TMV) replicase protein [22]. These miRNAs play a functional role in *N*-regulation because their overexpression was shown to compromise *N*-mediated resistance to TMV [22]. Another recent study conducted in *Solanum lycopersicum* showed that miR482, a 22 nt long conserved miRNA that targets dozen of CNLs, was down-regulated in response to unrelated viruses as well as to a bacterium that encode RNA silencing suppressors [21]. Interestingly, this phenomenon was associated with the derepression of some CNLs that are targeted by miR482, suggesting that pathogen-triggered suppression of RNA silencing likely derepresses a whole repertoire of immune receptors during infection that might contribute to plant immunity [21].

Recent findings have thus revealed a critical role of miRNA-directed phased siRNA production in controlling the expression of *R* gene transcripts in the context of pathogen infection. Nevertheless, the interplay between the dynamic regulation of the RNA silencing machinery involved in miRNA-directed secondary siRNA production and the post-transcriptional regulation of *R* gene transcripts that are targeted by these small RNA species remains unknown. In addition, whereas some intracellular immune-receptors have recently been characterized in basal defense as well as plant defense against a disarmed bacterium very little is known on the functional relevance of plant NLRs in PTI [21]. The present study addresses some of these important issues by studying the regulation of *RDR6* during antibacterial defense and the role of this silencing factor in the control of CNLs that are targeted by the Arabidopsis miR472, a miRNA related to miR482.

## Results

### Arabidopsis *RDR6* negatively regulates PTI responses

**Figure 1:**
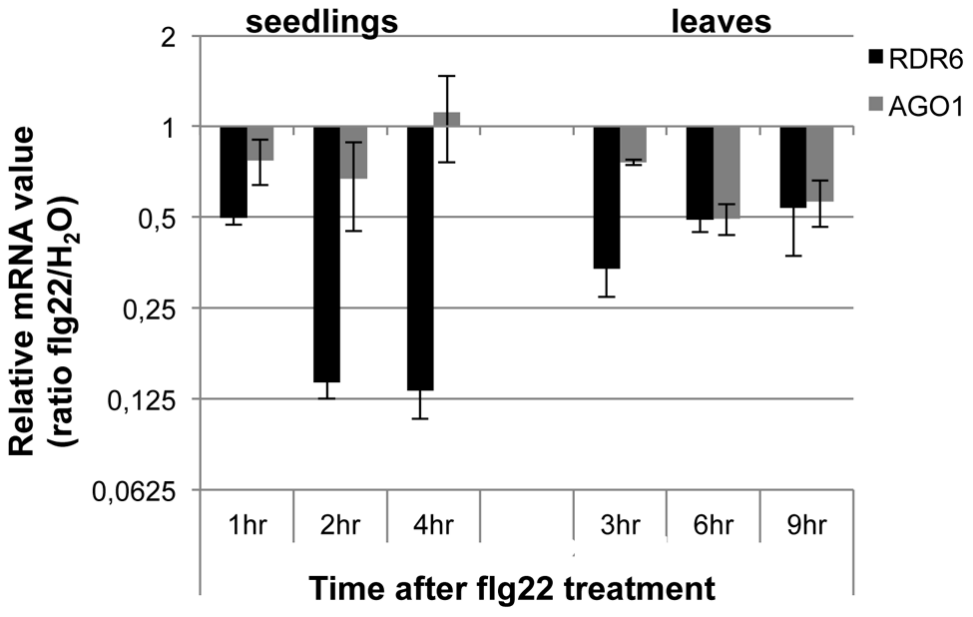
RDR6 and AGO1 mRNA levels rapidly decrease after flg22 treatment. Seedlings were treated for 1, 2 and 4-qPCR. Expression levels are relative to three reference genes (At2g36060; At4g29130; At5g13440). The Log_2_ of mRNA values are normalized to that of control WT seedlings or leaves plants treated or infiltrated with water. Error bars indicate standard deviation from technical repeats. Similar results were obtained in two independent experiments.

**Figure 2:**
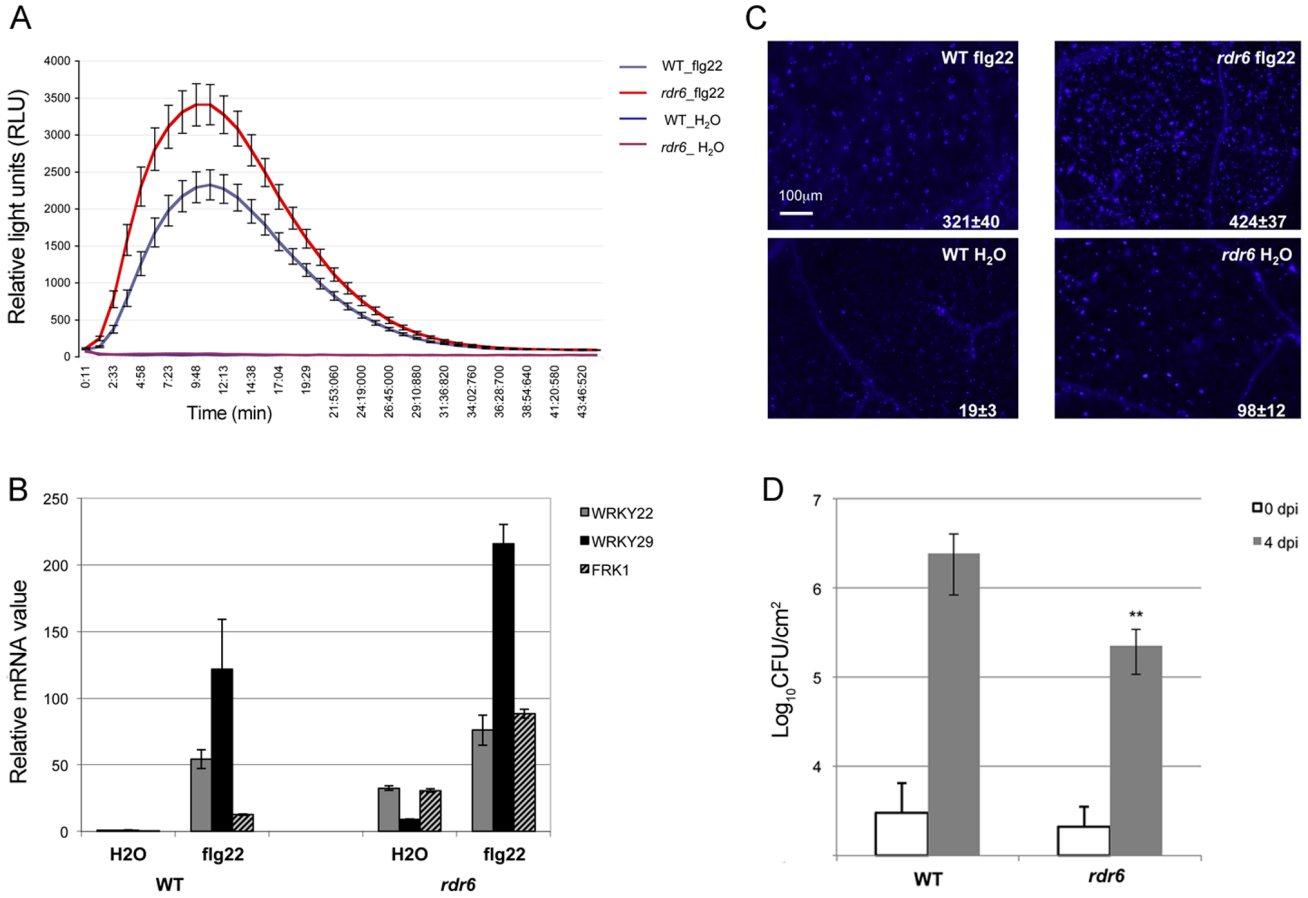
*RDR6* negatively regulates PTI responses. (**A**) H_2_O_2_-dependent luminescence upon H_2_O or flg22 (100 nM) treatment in WT and *rdr6-15* leaf discs. (**B**) Expression levels of PAMP-responsive mRNAs, FRK1, WRKY22 and WRKY29 detected by RT-qPCR in leaves treated with 100 nM flg22 or water for 4 hours. (**C**) Callose deposition upon H_2_O or flg22 (100 nM) treatment in WT and *rdr6-15* leaves blade at stage 7. Values are average ± se (standard error) with n = 25 to 30. (**D**) Bacterial growth in five- to six-week-old plants (WT or *rdr6*) 4 days after being sprayed (10^8^ CFU mL^−1^) with *Pto* DC3000. Values are average ± se of four leaf discs (n = 8). Wilcoxon test was performed to determine the significant differences between *rdr6* and WT plants. Asterisk “**” indicates statistically significant differences (P<0.01). Experiments were performed in two independent biological replicates with similar results.

Although ARGONAUTE 1 (AGO1) and DICER-LIKE 1 (DCL1) were previously shown to contribute to PTI [37], [38], their regulation during the plant innate immune response has not been determined. To get a first insight into the regulation of components of PTGS during plant defense, we examined the expression levels of well-characterized PTGS factors in multiple conditions known to trigger PTI responses (Genevestigator database: https://www.genevestigator.com). Results from this analysis revealed that RDR6, AGO1 and SUPPRESSOR OF GENE SILENCING 3 (SGS3) mRNAs [39] were all down-regulated, with RDR6 showing the highest difference (consistently more than 2-fold in the various conditions analyzed) (**Figures S1A, S1B**). Accordingly, Reverse-Transcriptase Quantitative Polymerase chain reaction (RT-qPCR) analyses revealed a significant decrease in RDR6 mRNA levels in Arabidopsis leaves and seedlings treated with the flagellin-derived peptide flg22 (**Figure 1**), with a decrease in RDR6 transcripts starting at 10 min in Arabidopsis elicited seedlings (**Figure S1C**). A similar effect was observed with the type-three secretion (TTS) defective mutant *Pto* DC3000 *hrcC* ^−^, which can elicit, but not suppress, PTI responses due to its inability to inject effector proteins within host cells (**figure S1C**). The PAMP-triggered dynamic regulation of RDR6 transcripts therefore suggested a potential role for RDR6 in orchestrating PTI responses. To test this idea, we first monitored the effect of the *rdr6-15* loss-of-function mutation on the production of reactive oxygen species (ROS), one of the earliest cellular responses following PAMP perception, which is known to orchestrate the establishment of different defensive barriers against biotrophic pathogens [40]. We observed a more pronounced flg22-triggered oxidative burst in the *rdr6* mutant as compared to WT-elicited plants (**Figure 2A**). However, given that the kinetics of flg22-triggered ROS production precedes the down-regulation of RDR6 transcripts in wild-type treated plants (**Figure 1**), these results suggest that the repression of RDR6 mRNAs is unlikely causative for this early PTI response. We also monitored the expression of PTI marker genes and found a primed induction of *Flg22 RECEPTOR KINASE 1* (*FRK1*) in the *rdr6*-elicited mutant (**Figure 2B**, [41]). Of note, induction of *FRK1* as well as the two other early PTI marker genes *WRKY22* and *WRKY29* was also moderately sensitized upon syringe infiltration of water in *rdr6*- *versus* WT-leaves (**Figure 2B**), suggesting that *RDR6* may additionally repress a wounding response caused by mechanical stress.

We further monitored the flg22-triggered formation of cell wall depositions of callose, a late PTI response that plays a critical role in the establishment of basal immunity [42], [43]. An increase in flg22-induced callose depositions was observed in the *rdr6-15* mutant as compared to WT plants, reinforcing a role for *RDR6* in repressing this late PTI response (**Figure 2C**). It is noteworthy that a higher number of callose deposits were also observed in mock-treated *rdr6-15* mutant *versus* WT plants, but not in untreated *rdr6-15* mutant leaves (data not shown), suggesting that *RDR6* may additionally prevent callose deposition upon wounding caused by syringe infiltration.

Natural surface openings, such as stomata, are important entry sites for bacterial plant pathogens such as *Pto* DC3000 and previous studies have shown that stomata closure plays an active role in limiting bacterial invasion as part of PTI responses [4]. Furthermore, *fls2* mutants were found to be more susceptible to *Pto* DC3000 upon spray inoculation, although no discernible phenotype was observed using classical syringe infiltration assay, which bypassed basal immunity present at the leaf surface [44]. Given that the *rdr6-15* mutant was sensitized for multiple flg22-triggered PTI responses, we reasoned that such silencing-deficient mutant might display enhanced resistance to *Pto* DC3000 upon spray inoculation. Consistent with this hypothesis, we found ∼10 times lower bacterial titer on *rdr6-15* mutant as compared to WT plants spray inoculated with *Pto* DC3000 (**Figure 2D**). Collectively, these data provide evidence that the RNA silencing factor *RDR6* acts as a negative regulator of basal immunity. These results also suggest that some positive regulators of plant defense are likely to be directly controlled by *RDR6*-dependent siRNAs.

### *RDR6*-dependent secondary siRNAs target a subset of mRNAs encoding CNL proteins

Besides generating siRNAs directed against viral-, transgene- and transposon-derived RNAs, *RDR6* is known to produce secondary 21 nt siRNAs from several endogenous loci including *TAS* genes [25], [26]. We thus searched for candidate defense gene transcripts that would be directly controlled by *RDR6*-dependent siRNAs. We used publicly available small RNA libraries derived from WT and *rdr6* mutant leaves and selected candidate genes with a significant reduced amount of 21 nt siRNAs in the *rdr6* as compared to the WT background. Using such criterion, we identified 75 loci that were likely targeted by *RDR6*-dependent siRNAs. Among those, 27 were previously annotated as *TAS* genes or tasiRNA targets. The remaining 48 protein-coding genes were enriched in GO categories ‘response to stress’ (http://bar.utoronto.ca/welcome.htm), (**Figure S2**), and include well-characterized *RDR6*-dependent targets such as AGO1, which is targeted by miR168-directed secondary siRNAs [45]. In addition, thirteen other candidate genes were annotated as miRNA targets and include multiple disease resistance gene transcripts that were previously identified as targets of miR472 (**Figures S3, S4**), a 22 nt long miRNA that is at least in part loaded into AGO1-RISC [35], [46]. These *R* genes are phylogenetically related to the functionally relevant disease resistance gene *RPS5*, which was previously characterized in ETI [47].

### *RDR6*-dependent siRNAs negatively regulate a subset of CNL transcripts both constitutively and during flg22 elicitation

**Figure 3:**
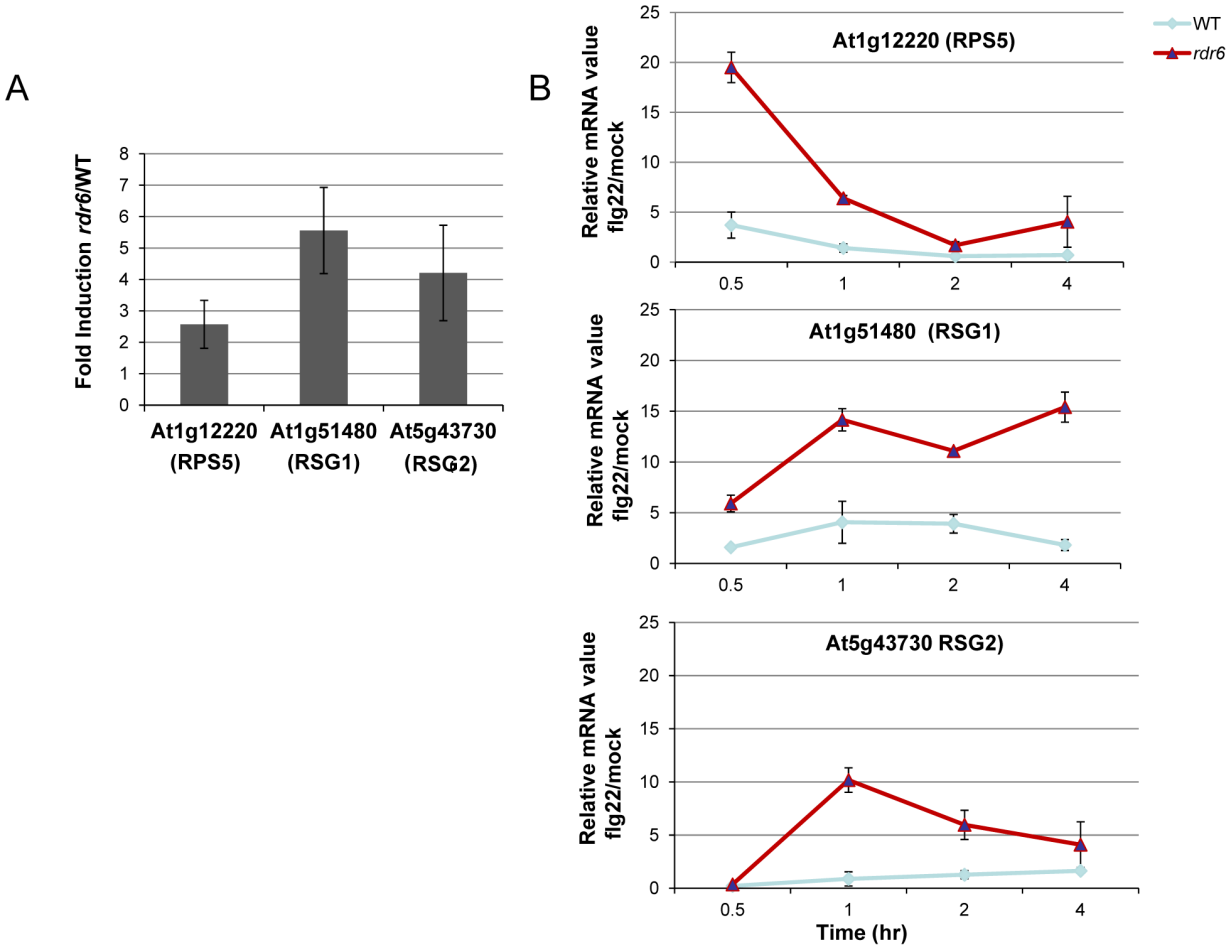
*RDR6*-dependent siRNAs negatively regulate a subset of CNL transcripts both constitutively and during flg22 elicitation. (**A**) The transcript levels of At1g12220 (RPS5), At1g51480 (RSG1) and At5g43730 (RSG2) were detected by RT-qPCR in untreated seedlings. Results of expression represent the ratio *rdr6*/WT. (**B**) Transcript levels of RPS5, RSG1 and RSG2 by RT-qPCR in seedling treated or not with flg22 (100 nM) at different time-points. Results represent the ratio of the values between samples treated with flg22 relative to H_2_O (mock) for *rdr6* and WT seedlings. Expression levels are always normalized to the same internal controls At2g36060, At4g29130, and At5g13440. Error bars indicate standard deviation from technical repeats. These experiments were performed in two biological replicates with similar results.

Given that At1g51480 and At5g43730 were among the CNL transcripts with the most matching secondary siRNAs (**Figure S4**), we decided to further characterize their regulation by *RDR6* in both naïve and flg22-challenged conditions. These candidate genes are referred to here as *Resistance Silenced Gene 1* (*RSG1*, At1g51480) and *Resistance Silenced Gene 2* (*RSG2*, At5g43730). We also included *RPS5* in this analysis, which was previously validated as miR472 target in Parallel Analysis of RNA Ends (PARE) datasets. We found a mild enhanced accumulation of these three candidate transcripts in unchallenged *rdr6* mutant as compared to non-treated WT seedlings (**Figure 3A**), suggesting that these mRNAs are weakly controlled by *RDR6*-dependent siRNAs in naïve conditions, presumably due to their low basal transcriptional level in unchallenged conditions as previously observed for several disease resistance genes [17], [48]. We next monitored the levels of these mRNAs upon flg22 treatment in both WT and *rdr6* mutant backgrounds. Whereas a mild increased induction of these transcripts was found in WT-elicited background, as observed in publicly available datasets (**Figure S5**), a 10- to 20-fold enhanced accumulation of these transcripts was obtained in the *rdr6*-elicited mutant seedlings, indicating cell priming in the absence of *RDR6*-dependent siRNAs (**Figure 3B**). These results therefore indicate that *RDR6*-dependent secondary siRNAs negatively regulate these CNL transcripts and that this post-transcriptional regulatory control is particularly relevant during PTI, when these disease resistance genes are presumably transcriptionally activated.

### MiR472 negatively regulates PTI responses and resistance against virulent *Pto* DC3000

**Figure 4:**
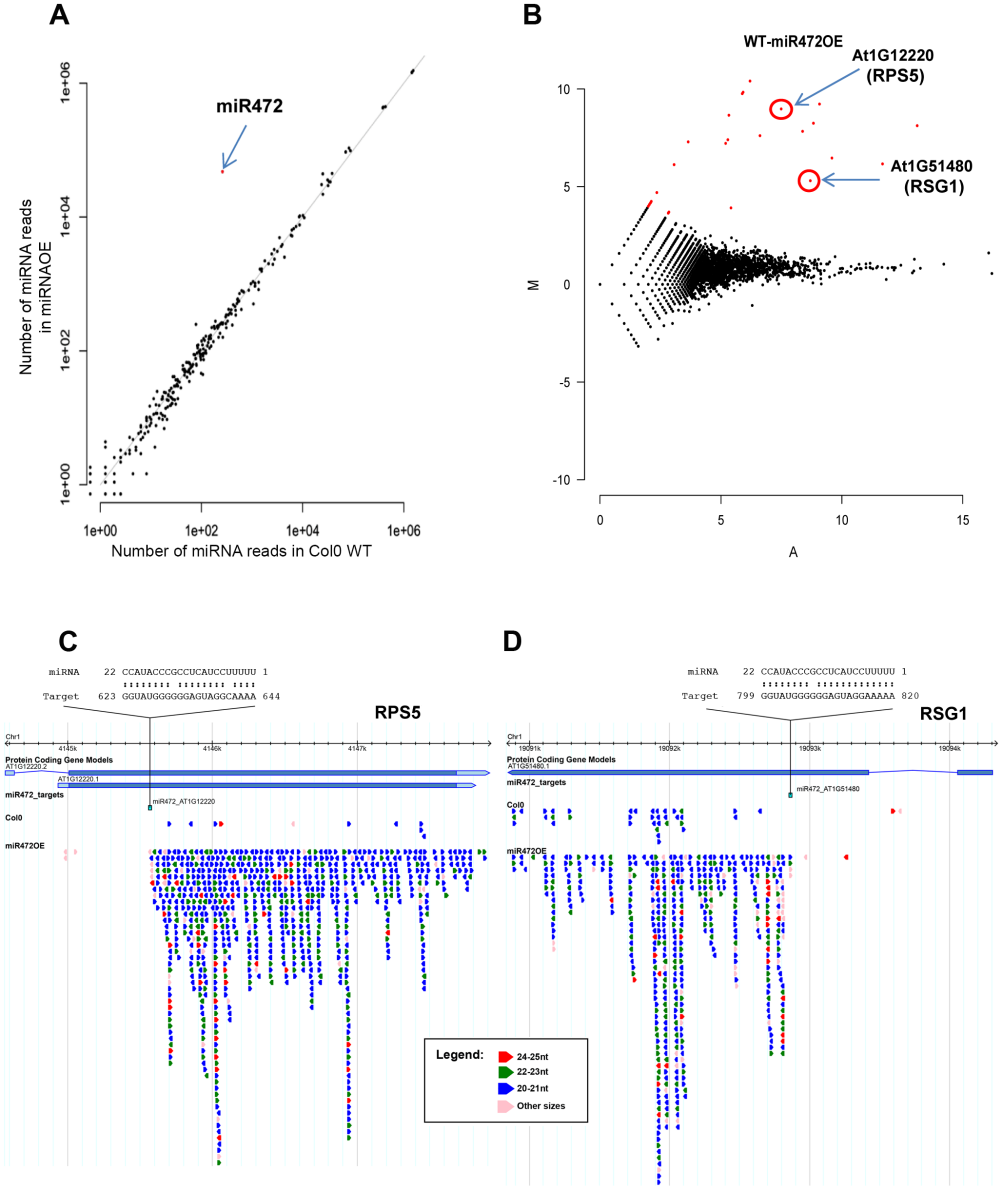
Overexpression of miR472 drastically enhances the accumulation of secondary siRNAs at multiple CNL transcripts. (A) Scatter plot representation of the number of reads corresponding to miRNA stem-loop loci (miRBase release 19) in WT and miR472OE mutant sRNA libraries. The number of reads was library size normalized. The red dot corresponds to miR472. (**B**) MA plot representation of the results obtained after differential analysis of 20–22 nt small RNAs accumulation from genes, between WT and miR472OE mutant. The y axis represents the log ratio (log_10_) of the library size normalized number of reads between the 2 datasets and the x axis the average number of reads in the two libraries. Genes with a significantly higher sRNAs accumulation in miR472OE library are shown in red. (**C**) Example of sRNAs accumulation in WT and miR4720E libraries along 2 CNL genes, RPS5 (AT1g12220) and RSG1 (AT1G51480). Genome browser representation of sRNA reads along. Each arrow corresponds to a specific sRNA sequence with a colour code corresponding to it length as indicated in the legend. Position and alignment of miR472 recognition sites are indicated. It is remarkable that the accumulation of siRNAs is observed downstream the cleavage site of miR472.

Given that miR472 was shown to target the above CNL mRNAs and to initiate the production of *RDR6*-dependent secondary siRNAs at these loci [20], [21], [22], we next characterized the role of this particular miRNA in the regulation of these candidate CNL transcripts as well as other orphan targets. For this purpose, we first transformed Arabidopsis with a construct containing *AtmiR472* driven by the strong *Cauliflower Mosaic Virus* (*CaMV*) 35S promoter and selected a reference line (referred to as miR472OE line) exhibiting high miR472 accumulation compared to WT (**Figure 4A**). This line displayed a 25% and 30% reduction in the accumulation of RPS5 and RSG1 transcripts, respectively (**Figure S6**), providing further evidence that miR472 targets these CNL mRNAs in unchallenged conditions. Furthermore, genome-wide small RNA deep sequencing analyses revealed a drastic enhanced accumulation of secondary siRNAs at the 3′ ends of miR472 target sites for RPS5, RSG1 and RSG2 mRNAs as well as for 16 other CNL transcripts in the miR472OE line as compared to wild type seedlings (**Figures 4B, 4C** **, S7**). It is noteworthy that no siRNA were identified upstream the miR472 target site, which is in agreement with the rapid degradation of this region after miRNA-guided cleavage [49]. Furthermore, normal levels of tasiRNAs were identified in miR472OE as compared to WT seedlings (**Figure S8**), proving evidence that the enhanced accumulation of CNL-derived secondary siRNAs are not due to a general activation of the RDR6-dependent pathway in this transgenic line. Collectively, these results strongly reinforce a role for miR472 in initiating the biosynthesis of *RDR6*-dependent secondary siRNAs at our candidate CNL transcripts and revealed additional CNLs that are directly targeted by this regulatory process including the other functionally relevant disease resistance gene *SUMM2* (**Figure S9**) [50].

**Figure 5:**
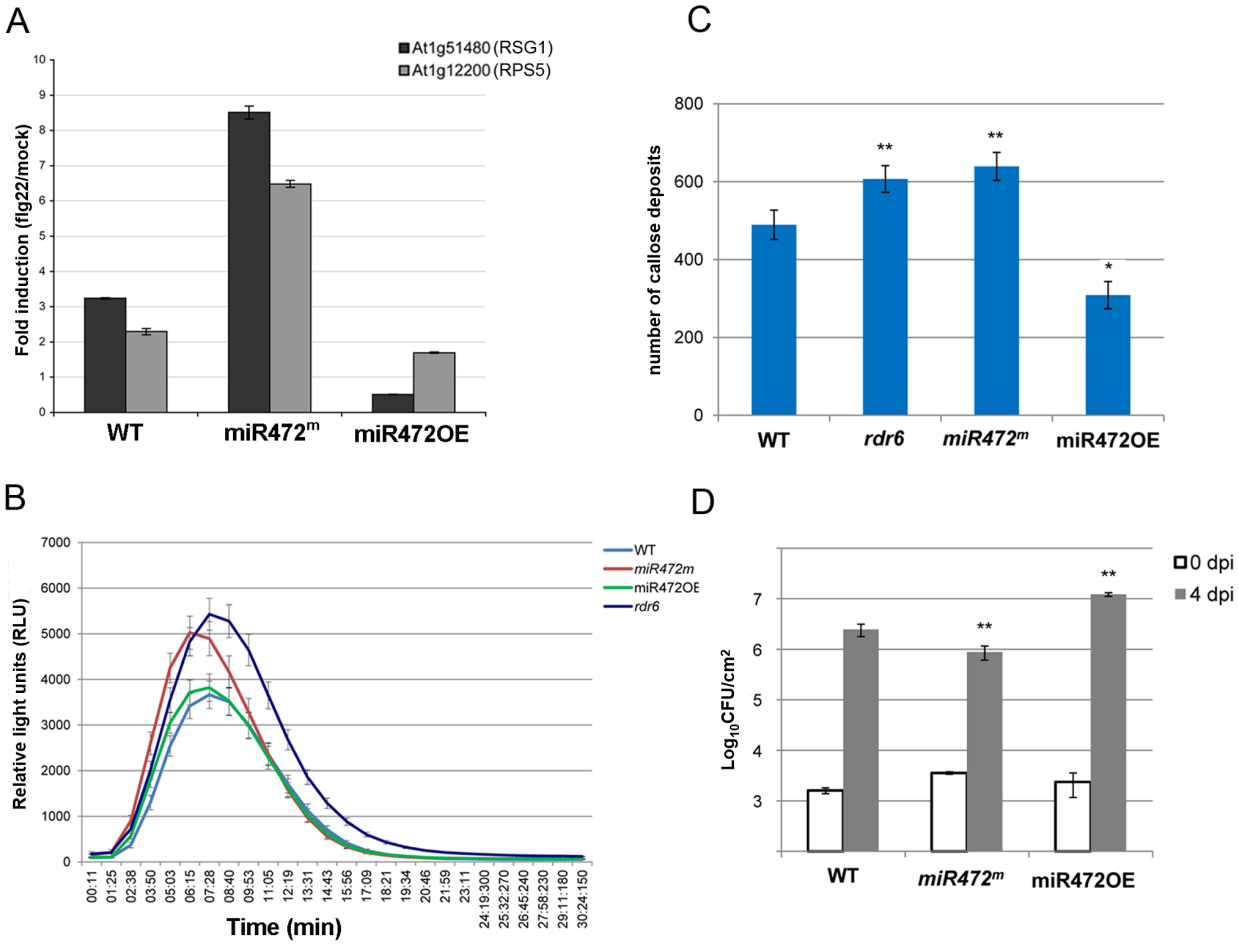
*MiR472* negatively regulates PTI responses and resistance against virulent *Pto* DC3000. (**A**) Expression levels of At1g12220 and At1g51480 detected by RT-qPCR in WT, miR472OE (overexpressor) and *miR472^m^* (mutant) seedling treated with either H_2_O or flg22 (100 nM) for 2 hours. (**B**) H_2_O_2_-dependent luminescence induced by flg22 (100 nM) in WT, miR472OE and miR472^m^ leaf discs. (**C**) Callose deposition induced by flg22 (100 nM) in WT, miR472OE and miR472^m^ leaves. (**D**) Bacterial growth in five- to six-week-old plants from WT, miR472OE and *miR472^m^* infiltrated with *Pto* DC3000 (2×10^5^ CFU mL^−1^). For C and D values are average ± se of four leaf discs (n = 8). Wilcoxon test was performed to determine the significant differences as compared to *rdr6* plants. Asterisks “*” and “**” indicate statistically significant differences at a P value<0.05 and <0.01 respectively. These experiments were performed in two biological replicates with similar results.

We next analyzed the mRNA accumulation of two candidate CNLs in the miR472OE line challenged with flg22. Flg22-triggered induction of RPS5 and RSG1 mRNAs was significantly impaired in miR472OE line as compared to WT-elicited control (**Figure 5A**), supporting a role for miR472 in regulating the accumulation of these targets during flg22 elicitation. We also examined different PTI features in the miR472OE reference line by monitoring ROS production and callose deposition upon flg22 treatment. While this transgenic line displayed a normal flg22-triggered ROS production as compared to WT-elicited control (**Figure 5B**), we found a reduced number of flg22-induced callose deposits relative to WT-treated plants (**Figure 5C**), indicating that the miR472OE reference line is altered in the latter PTI response. It is noteworthy that similar PTI phenotypes were observed in another independent transgenic line overexpressing miR472 (**Figure S10**).

To get further insights into the role of miR472 in the regulation of CNL transcripts and PTI responses, we further characterized a transgenic line carrying a T-DNA insertion within the promoter of the *AtmiR472* locus (Salk_087945, referred to as *miR472^m^*). This line displayed a drastic decrease in the accumulation of the mature form of miR472 relative to the levels of this miRNA in WT background (**Figure S11**). Furthermore, a primed induction of RPS5 and RSG1 transcripts was found in the *miR472^m^* line relative to WT background treated with flg22 (**Figure 5A**), supporting a role for miR472 in repressing mRNA accumulation of these CNL mRNAs during flg22 elicitation. Further phenotypic analyses in this line revealed a more pronounced flg22-induced ROS production and callose deposition, thereby mimicking the primed PTI responses observed in the *rdr6*-elicited mutant (**Figures 5B, 5C**). We thus conclude that miR472 and *RDR6*-dependent secondary siRNAs regulate PTI responses likely by targeting a whole repertoire of CNL transcripts.

Finally to determine the role of miR472 in basal resistance, we inoculated the virulent *Pto* DC3000 strain on miR472OE and *miR472^m^* lines and monitored bacterial titers in these genetic backgrounds as compared to WT-infected control. We found an increased *Pto* DC3000 titer in the miR472OE line, and, conversely, a reduced growth of this bacterium in the *miR472^m^* line as compared to WT-infected control (**Figures 5D** **, S10**). These results indicate that miR472 not only represses PTI responses but also negatively regulates basal resistance against *Pto* DC3000. These results also suggest that a subset of CNLs, which are targeted by miR472 and *RDR6*-dependent secondary siRNAs, may control basal resistance against *Pto* DC3000.

### *MiR472* and *RDR6* negatively regulate *RPS5*-mediated resistance

**Figure 6:**
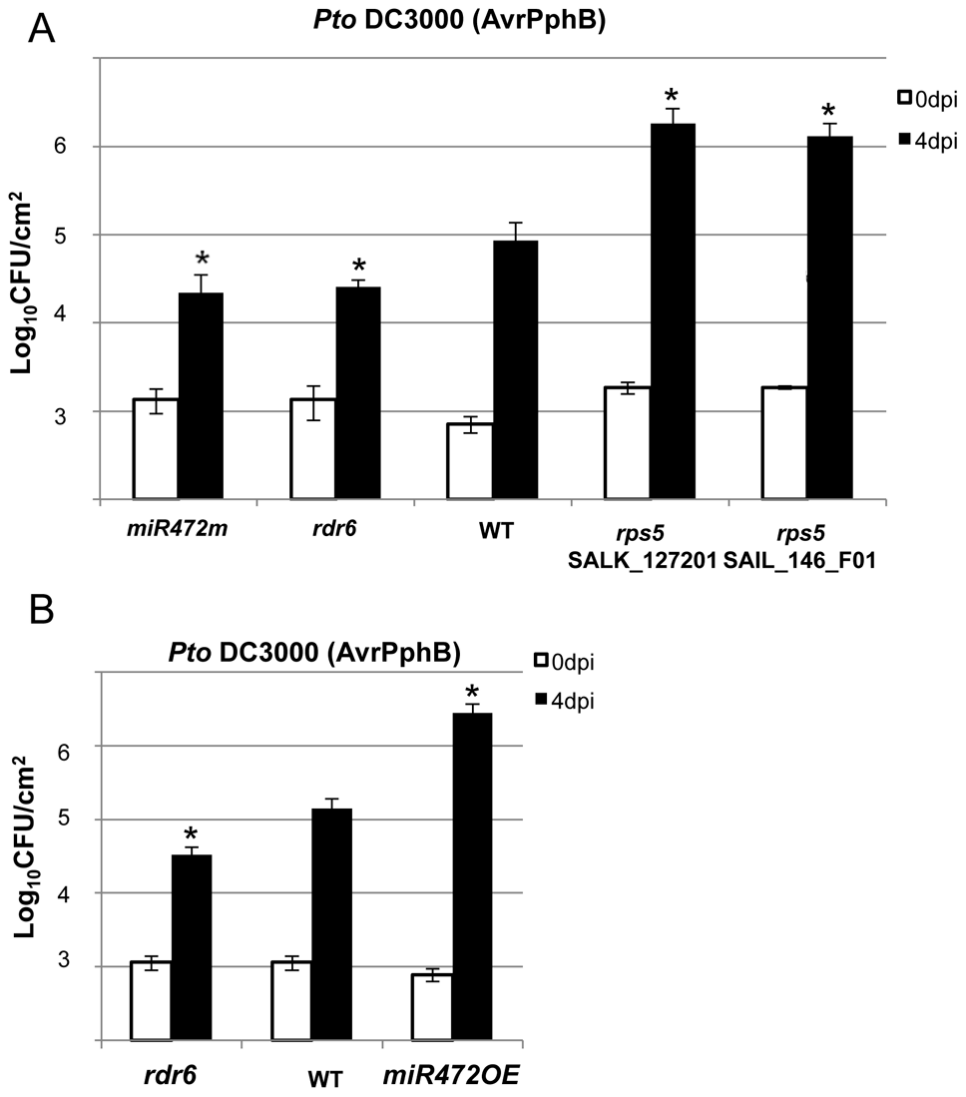
*MiR472* ^m^ and *rdr6* are more resistant to *Pto* DC3000 (AvrPphB). (**A**) Bacterial growth in five- to six-week-old plants from WT, *rdr6* and *miR472^m^* syringe-infiltrated with *Pto* DC3000 AvrPphB (2×10^5^ CFU mL^−1^). Values are average ± se of four leaf discs (n = 8). Wilcoxon test was performed to determine the significant differences as compared to WT plants. As a positive control the susceptible mutant *rps5* (two independent mutant alleles SalK_127201 and SAIL_146_F01) has been used as control. Asterisk “*” indicates statistically significant differences at a P value<0.05. (**B**) Bacterial growth in five- to six-week-old plants from WT and miR472OE lines syringe-infiltrated with *Pto* DC3000 AvrPphB (2×10^5^ CFU mL^−1^). Values are average ± se of four leaf discs (n = 8). Wilcoxon test was performed to determine the significant differences as compared to WT plants. These experiments were performed in two biological replicates with similar results.

The effective targeting of RPS5 mRNAs by miR472 and RDR6-dependent secondary siRNAs (**Figure 4B, 4C**), together with the well-characterized role of RPS5 in recognizing the bacterial effector AvrPphB and mounting ETI [47], prompted us to investigate the role of *miR472* and *RDR6* in *RPS5*-mediated resistance. For this purpose, the *rdr6-15* and *miR472^m^* lines were first inoculated with a *Pto* DC3000 strain carrying AvrPphB and bacterial titers were monitored at 4 days post-inoculation. Results from these analyses indicated a significant enhanced *RPS5*-mediated resistance in both *rdr6* and *miR472m* as revealed by lower bacterial titers in these mutants as compared to WT-infected plants (**Figure 6A**), which is consistent with the enhanced accumulation of RPS5 transcripts in these mutant backgrounds (**Figures 3A, 3B** **,** **5A**). Of note, this phenomenon was specific to *RPS5*-mediated resistance, because no phenotype was observed upon inoculation of *rdr6* and *miR472m* lines with *Pto* DC3000 expressing AvrRpt2, a bacterial effector that is recognized by another CNL that is not targeted by miR472 (**Figure S12**, [13]). We next inoculated the *Pto* DC3000 (AvrPphB) strain on the miR472OE reference line and monitored bacterial titers as well as disease symptoms at 4 days post-inoculation. Interestingly, we found a significant enhanced *Pto* DC3000 (AvrPphB) titer in the miR472OE line as compared to WT-infected plants (**Figure 6B**), which was associated with a rescue of both chlorotic and necrotic disease symptoms in this transgenic plants (data not shown), thereby mimicking the phenotypes observed in *rps5* loss-of-function mutants (**Figure 6A**, [47]). We conclude that overexpression of *miR472* is sufficient to compromise *RPS5*-mediated resistance, which is consistent with the reduced levels of RPS5 mRNAs in this transgenic line (**Figures 5A** **, S6**). Collectively, these results indicate that *miR472* and *RDR6* negatively regulate not only PTI but also RPS5-mediated resistance, suggesting a critical role for *RPS5* and other *CNLs* in basal and race-specific immunity.

### Proper chaperoning of NB-LRRs is required for the enhanced basal immunity and *RPS5*-mediated resistance phenotypes observed in the *rdr6* mutant

**Figure 7:**
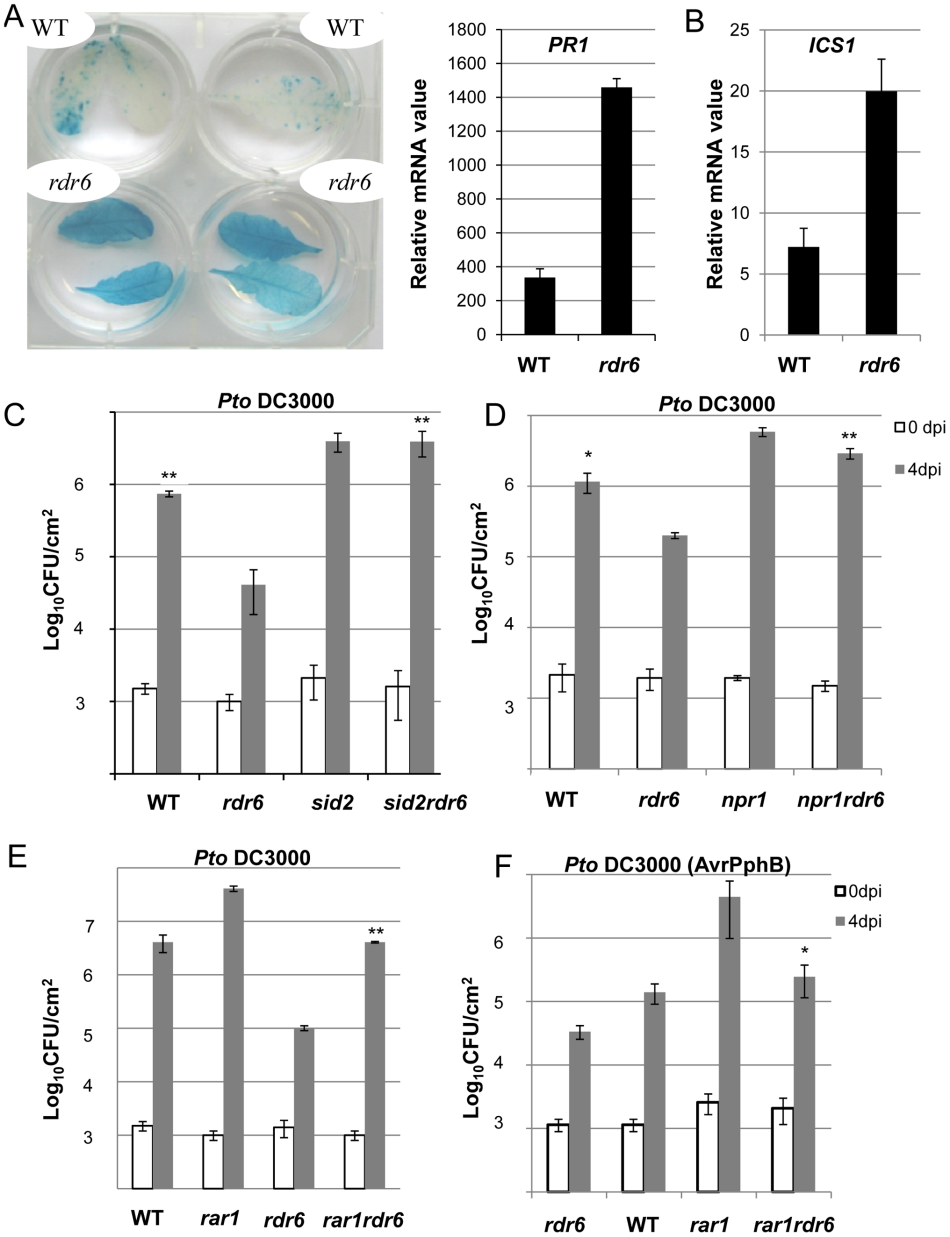
Enhanced basal resistance towards *Pto* DC3000 observed in the *rdr6* mutant requires SA and proper chaperoning of NLRs. (**A**) β-glucuronidase (GUS) activity in plants PR1p:GUS and *rdr6* PR1p:GUS plants reporting PR1 transcriptional activity in WT and *rdr6*-15 mutant, respectively. (**B**) The transcript level of PR1 and ICS1 were detected by RT-qPCR. Error bars indicate standard deviation from technical repeats. Expression levels are normalized to the same internal controls At2g36060, At4g29130, and At5g13440. (**C**) Bacterial growth in five- to six-week-old plants from WT, single *rdr6-15* and *sid2-2* mutants or double *rdr6-sid2* mutant infiltrated with *Pto* DC3000 (2×10^5^ CFU mL^−1^). (**D**) Bacterial growth in five- to six-week-old plants from WT, simple *rdr6-15* and *npr1-1* mutants or double *rdr6-npr1* mutant infiltrated with *Pto* DC3000 (2×10^5^ CFU mL^−1^). (**E**) Bacterial growth in five- to six-week-old plants from WT, single (*rdr6-15, rar1-21*) or double mutant (*rdr6-rar1*) infiltrated with *Pto* DC3000 (2×10^5^ CFU mL^1^). F) Bacterial growth in five- to six-week-old plants from WT, single (*rdr6-15, rar1-21*) or double mutant (*rdr6-rar1*) infiltrated with *Pto* DC3000 (AvrPphB) (2 10^5^ CFU mL^1^). For **C, D, E** and **F** values are average ± se of four leaf discs (n = 8). Wilcoxon test was performed to determine the significant differences between *rdr6* and double mutant plants. Asterisks “**” and “*” indicate statistically significant differences at a P value<0.01 and <0.05 respectively. Experiments were performed in two independent biological replicates with similar results.

The predicted target site of miR472 is embedded within a region encoding the P-loop domain, which is highly conserved in a large repertoire of CNL disease resistance proteins [9]. It is therefore likely that multiple CNLs are controlled by this particular miRNA and, in agreement, 19 CNL transcripts were experimentally validated as miR472 targets in Arabidopsis seedlings overexpressing *miR472* (**Figures 4** **, S7**). This suggests that the enhanced basal resistance phenotype observed in the *rdr6* and *miR472^m^* mutants might not only be due to the constitutive expression and/or primed induction of the few CNLs that have been characterized in these mutant backgrounds (e.g. RPS5), but also likely to multiple other relatives that are targeted by these small RNAs, rendering the functional characterization of these CNLs challenging. To circumvent this issue, we first introduced, in the *rdr6* mutant background, mutations that abolish CNL-mediated signaling, and subsequently monitored *Pto* DC3000 titer in these double mutant backgrounds. Since several CNLs are known to trigger SA-signaling/biosynthesis [51], including RPS5 [52], we hypothesized that the SA-dependent defense response might be constitutive in the *rdr6* mutant background. Consistent with this idea, we found a constitutive expression of the SA-dependent marker gene *PATHOGENESIS-RELATED 1* (*PR1*) and the *ISOCHORISMATE SYNTHASE1 (ICS1)* (**Figures 7A, 7B**) [51], [53], as well as an enhanced resistance to *Pto* DC3000, in the *rdr6* mutant as compared to WT control (**Figures 7C, 7D, 7E, 7F**). Importantly, this increased resistance to *Pto* DC3000 was abolished by introducing mutations that compromise SA-biosynthesis (the *sid2-2* mutation, [53]) or SA-signaling (the *npr1-1* mutation, [54]) in the *rdr6-15* mutant background (**Figures 7C, 7D**). These results therefore indicate that the enhanced basal resistance achieved in the *rdr6* mutant relies on the constitutive activation of the SA-dependent defense response, which might be initially triggered by the enhanced accumulation of CNLs that are no longer controlled by *RDR6*-dependent secondary siRNAs in this mutant background.

To get further insights into the role of these CNLs in the enhanced basal resistance phenotype observed in the *rdr6* mutant, we took advantage of the property of the REQUIRED FOR MLA12 RESISTANCE (RAR1) protein. RAR1 is part of a molecular chaperone complex, containing HEAT SHOCK PROTEIN 90 (HSP90) and SUPPRESSOR OF G-TWO ALLELE OF SKP1 (SGT1), and plays a major role in NLR protein stability and activity [55]–[65]. Importantly, the steady-state accumulation of several CNL proteins, including RPS5, was shown to be dramatically impaired in *rar1* loss-of-function mutants [58], [64]–[68]. We thus reasoned that by introducing a *rar1* loss-of-function mutation in the *rdr6* mutant background, we would destabilize CNL proteins whose cognate mRNAs are targeted by RDR6-dependent siRNAs, and therefore potentially restore disease susceptibility. Consistent with this hypothesis, we found that the increased resistance achieved in the *rdr6* mutant was abolished in the *rdr6*-*rar1* double mutant (**Figure 7E**). It is noteworthy that an enhanced *Pto* DC3000 titer was also found in the single *rar1* and double *rdr6-rar1* mutants as compared to WT control, indicating that *RAR1* contributes to basal resistance as previously reported [64]. Given that *RDR6* was found to negatively regulate *RPS5*-mediated resistance (**Figure 6**), we also monitored *Pto* DC3000 (AvrPphB) titer in the single *rdr6* mutant as compared to the *rdr6-rar1* double mutant. Results from these analyses indicated that the enhanced *RPS5*-mediated resistance observed in *rdr6* mutants was partially compromised in the *rdr6-rar1* mutant (**Figure 7F**). Collectively, these results indicate that the increased basal and specific resistance observed in the *rdr6* mutant is dependent on the proper chaperoning of CNL proteins (e.g. RPS5), and might therefore be due to the enhanced accumulation of CNL proteins whose cognate mRNAs are no longer controlled by endogenous secondary siRNAs in this silencing-defective mutant.

## Discussion

### Arabidopsis *RDR6* negatively regulates PTI, basal resistance, SA-dependent defense and *RPS5*-mediated resistance

*RDR6* has been clearly implicated as a positive regulator of virus and viroid resistance. Indeed silencing of *RDR6* in *Nicotiana benthamiana* results in hyper-susceptibility to some viruses and viroids [29], [30]. Moreover, *in situ* hybridization shows that viruses and viroids invade floral and vegetative meristems of *N. benthamiana rdr6* RNAi plants [69], [70]. Here, by combining microbiological, genetic, genomic and molecular techniques, we demonstrate that *RDR6* also acts as a negative regulator of PTI, basal defense as well as *RPS5*-mediated resistance. Indeed, we first showed that knock-out of *RDR6* renders the plants more resistant to the hemibiotrophic pathogen *Pto* DC3000 and to the avirulent *Pto* DC3000 (AvrPphB) strain (**Figures 2D** **,** **6A** **,** **7C, 7D, 7E**). Furthermore, classical PTI responses such as ROS production, mRNA accumulation of PAMP-response genes as well as callose deposition were increased in *rdr6* plants as compared to WT plants upon flg22 treatment (**Figures 2A, 2B, 2C**) [71]. Our results are thus in sharp contrast with the previously reported PTI phenotypes observed in *ago1* loss-of-function mutants [38]. Why is there such a discrepancy between these PTGS-defective mutant phenotypes during PTI? One would argue that AGO1 is not only involved in the siRNA pathway but also in the canonical miRNA pathway. AGO1 impairment has thus additional consequences on the action of several miRNAs necessary for PTI [38], [72], thereby leading to the previously reported compromised PTI responses in *ago1* loss-of-function mutants such as in other miRNA-defective mutants [37], [38]. It is also possible that *RDR6*-derived siRNAs that target disease resistance genes may not only be loaded into AGO1-RISC but also into other as-yet unknown AGO-RISCs, thereby contributing in part to the post-transcriptional regulation of CNLs in an AGO1-independent manner.

We also observed a constitutive activation of the SA defense marker gene *PR1* and an enhanced expression of *ICS1* in the *rdr6* loss-of function mutant (**Figures 7A, 7B**). To examine the involvement of the SA-dependent defense in the enhanced disease resistance phenotype observed in *rdr6* mutant, the *rdr6-15* mutation was combined with the *sid2-2*, a loss-of-function mutation in *ICS1* also referred to as *SID2* [53]. Inactivation of *ISC1*/*SID2* abolishes *rdr6* resistance to *Pto* DC3000 and similar results were obtained in the *npr1* mutant, which is impaired in SA signaling [54] (**Figures 7C, 7D**). Therefore, the SA-dependent defense pathway plays a critical role in the enhanced basal resistance phenotype observed in the *rdr6* mutant. Such constitutive SA-dependent defense response might result from a derepression of a subset of CNL transcripts (e.g. RPS5 mRNAs) that are no longer regulated by secondary siRNAs in this silencing-defective mutant. Additionally, it may result from the post-translational activation of R proteins that would be constitutively present in a protein complex with RDR6 and active in the absence of this silencing factor, as observed in classical ‘guardee’ mutants [10]. Further investigations will be necessary to address these possibilities. Moreover, additional experiments will be required to determine whether the constitutive SA-dependent defense response observed in the *rdr6* mutant is linked with the mild constitutive PTI responses in this silencing-defective mutant or whether both processes remain independent.

We observed a higher expression of PAMP-response marker genes in unchallenged *rdr6* mutant as compared to WT seedlings and a significant hyper-induction of FRK1 in the *rdr6*-elicited mutant (**Figure 2B**). Furthermore, a more pronounced callose deposition as well as ROS production were observed in the *rdr6* mutant challenged with flg22 as compared to WT-elicited seedlings (**Figures 2A, 2C**), indicating that this silencing-defective mutant is in a physiological situation known as “primed” state [73]. Those results also indicate that *RDR6* encodes a novel negative regulator of PTI and further reinforce the idea that PTI is under a tight negative regulatory control as previously reported [2], [74], [75], [76]. Interestingly, an analogous RNA silencing-dependent regulatory phenomenon has been recently described in the transcriptional control of a disease resistance gene during PTI [24]. In this case, flg22 was shown to trigger the repression of a subset of RNA-directed DNA methylation factors and this process was associated with TGS release and with the transcriptional activation of this immune receptor, which is targeted by siRNA-directed DNA methylation in its promoter region [24]. Although RDR6 mRNAs were down-regulated in response to flg22 (**Figure 1**), it remains to be tested whether this molecular effect could be accompanied with a decrease in RDR6 protein levels as well as an eventual global release of RDR6-silencing as part of PTI responses.

### Arabidopsis *RDR6* and *miR472* repress basal resistance and *RPS5*-mediated resistance likely by controlling a subset of CNLs at the post-transcriptional level

How does *RDR6* repress PTI, basal resistance and *RPS5*-mediated resistance? A first *in silico* analysis of small RNA populations derived from *rdr6* mutant as compared to wild-type leaf samples allowed us to identify *R* gene mRNA candidates that are targeted by *RDR6*-dependent secondary siRNAs (**Figure S4**). However, the low abundance of secondary siRNAs in the majority of cases limited the identification of such *miR472/RDR6* targets. By contrast, the use of our transgenic line overexpressing *miR472* was instrumental in identifying with confidence 19 *bona fide* CNL target transcripts that contain the miR472 recognition sites as well as a large number of secondary siRNAs located downstream of their miR472-guided cleavage site (**Figures 4** **, S9**). Among these candidates, we have identified RPS5 and SUMM2 transcripts, which encode functionally relevant disease resistance proteins with well-characterized role in ETI [47], [50], [56]. These results therefore suggested that the *miR472/RDR6*-silencing pathway inhibits the accumulation not only of disease resistance gene transcripts encoding R proteins required for PTI and basal resistance but also of transcripts encoding immune receptors required for ETI. This implicates *miR472* and *RDR6* in a central regulatory pathway that modulates both ETI and PTI responses. Consistent with this, we found that *RDR6* and *miR472* act not only as negative regulators of PTI and basal immunity but also as repressors of *RPS5*-mediated resistance (**Figure 6**). In addition, the use of the *rar1* mutant, which destabilizes disease resistance proteins including RPS5 [58], [59] was useful to provide genetic evidence that the enhanced disease resistance phenotypes observed in the *rdr6* mutant is likely the result of a higher accumulation of NB-LRR proteins in this silencing-defective mutant (**Figure 7**).

The present work also provides genetic evidence that *miR472*- and *RDR6*-dependent secondary siRNAs efficiently control the steady state levels of three CNL transcripts. Indeed, we first showed that RPS5, RSG1 and RSG2 mRNAs were moderately up-regulated in untreated *rdr6* mutant and significantly hyper-induced in this silencing-defective mutant challenged with flg22 (**Figure 3B**). Accordingly, a lower level of RPS5 and RSG1 mRNAs was detected in the miR472OE line (**Figure S6**) and a compromised induction of these CNL transcripts was also observed in this transgenic line challenged with flg22 (**Figures 5A, 5B, 5C**). Conversely, the *miR472* knock-down line displayed higher accumulation of CNL mRNAs, which was associated with increased PTI responses (**Figure 5**), therefore mimicking the phenotypes observed in the *rdr6* mutant (**Figure 2**). Collectively, these results indicate that both Arabidopsis *RDR6* and *miR472* negatively regulate the steady state levels of these candidate CNL transcripts in normal growth conditions and during PTI, although these effects appear more pronounced during the elicitation possibly due to the concomitant transcriptional activation of these *R* genes as previously demonstrated for other biotic stress responsive disease resistance genes [17], [18], [48].

**Figure 8:**
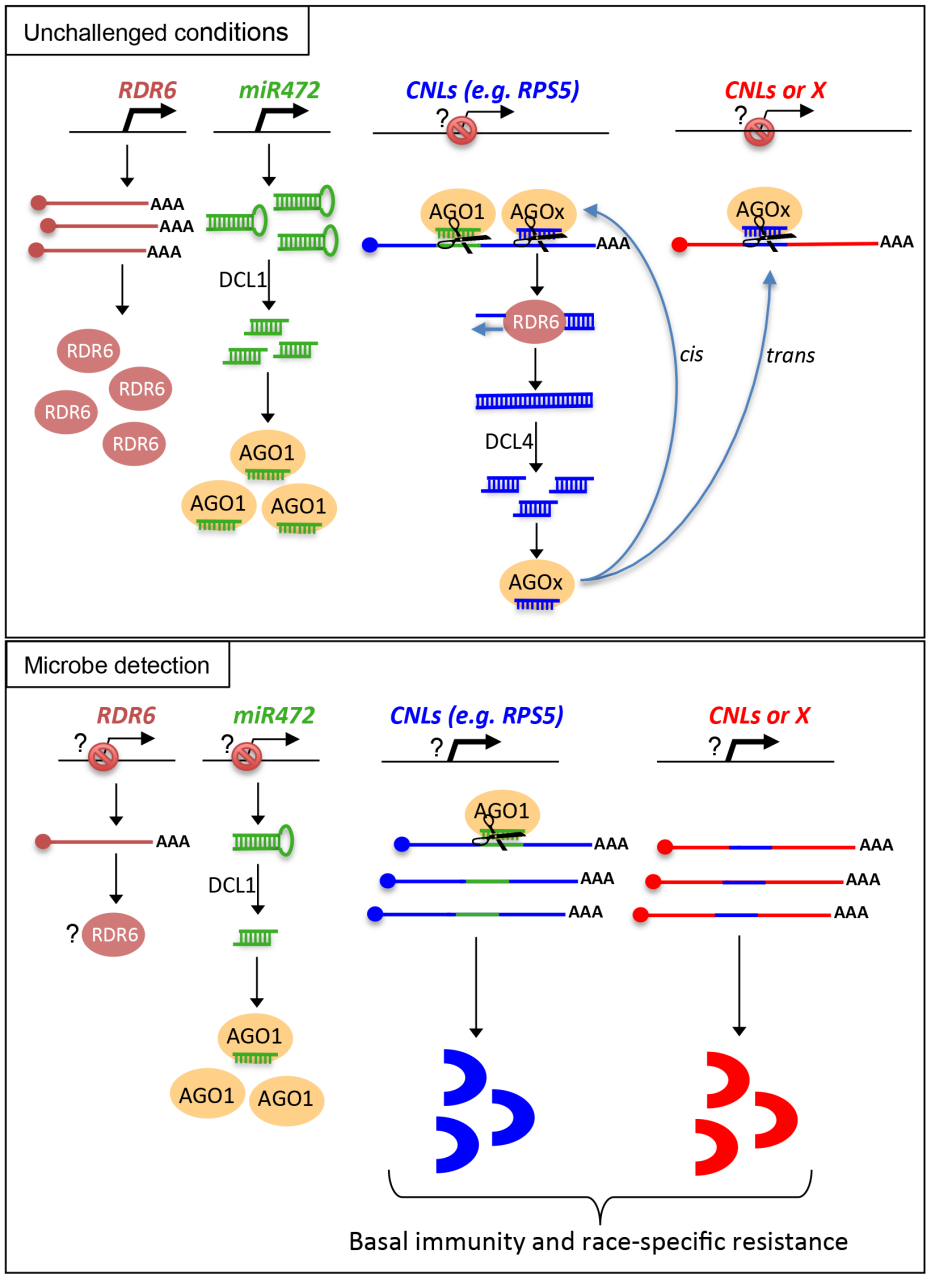
Schematic representation illustrating the relationship between miR472/RDR6 through CNL regulation during Arabidopsis immunity.

Based on these results, we propose a model, which integrates the contribution of the *miR472*/*RDR6*-dependent PTGS pathway in plant immunity (**Figure 8**). In unchallenged conditions, both *miR472* and *RDR6* are constitutively expressed and negatively regulate a subset of CNL mRNAs at the post-transcriptional level (**Figure 8**). MiR472 guides cleavage of RPS5, RSG1, RSG2 and at least 16 other CNL transcripts that carry miR472 recognition sites and RDR6 uses 3′ cleavage products as substrates to generate dsRNAs that are presumably processed by DCL4 into 21 nt siRNAs (**Figure 8**). These secondary siRNAs can act in *cis* by guiding mRNA degradation of the CNL transcripts from which they are produced, but also likely in *trans* presumably by targeting CNLs as well as unrelated mRNAs that display sequence complementary to these small RNA species as was recently suggested in tomato ([21], **Figure S7**).

It is also likely that the genes encoding the above immune receptors remain at a transcriptionally inactive state in unchallenged conditions as demonstrated for several other disease resistance genes [17], [48]. In this case, the concomitant low basal transcriptional expression of CNLs and the *miR472/RDR6*-dependent post-transcriptional regulatory process would effectively deplete immune receptor mRNAs in the absence of pathogens, thus preventing an autoimmune response that would have detrimental consequences on plant fitness [1], [77]. This is reminiscent of recent findings on other 22 nt miRNAs/secondary siRNAs that target NLR transcripts in different plant species [20], [21], [22], as well as with the observation that the production of siRNAs at the disease resistance *RPP4* cluster repress basal expression of several *R* gene transcripts within this cluster and likewise prevent constitutive activation of the SA-dependent defense pathway [18]. Our model also suggests that the mature form of miR472 is down-regulated during PTI, as a 4-fold decrease in the accumulation of this microRNA was observed in small RNA libraries generated by Li et al [38] upon flg22 treatment, which was confirmed in Arabidopsis leaves and seedlings treated with flg22 (**Figure S13**). We thus propose that upon pathogen detection, and perhaps also perception of non-adapted microbes, microbe-associated molecular patterns trigger the down-regulation of *miR472*, which in concert with the eventual transcriptional activation of CNLs, may contribute to the transient enhanced accumulation of CNL mRNAs/proteins at an early phase of the elicitation (**Figure 8**). This gene regulatory mechanism may also be reinforced by the down-regulation of *RDR6*-dependent silencing pathway as suggested by the rapid repression of RDR6 mRNAs during PTI (**Figure 1**). At a later phase of the elicitation, we propose that this double post-transcriptional layer of regulation mediated by *miR472* and *RDR6* likely trigger a robust resilencing of these CNL transcripts to prevent a sustained activation of the plant immune response.

### Functional relevance of disease resistance proteins in both ETI and PTI responses

Although R proteins have been extensively characterized in ETI [10], there is increasing evidence that these immune receptors can also contribute to basal defense as well as PTI responses in plants [78]. For example, a compromised basal resistance to virulent *Pto* DC3000 was previously reported in a *rar1* loss-of-function mutant [64], and confirmed in the present study (**Figure 7E**), suggesting that plant *NLRs* contribute to basal immunity. More recently, a subclade of CNL proteins, characterized as ‘helper NB-LRR’, where not only required for ETI but also for basal resistance and this process was independent of their P-loop motifs [79]. Importantly, these CNLs additionally regulate PAMP-triggered SA-accumulation in response to a disarmed *P. syringae* strain, which provides evidence that plant *NLRs* contribute to PTI [79]. Nevertheless, these CNLs do not control early events of PTI responses triggered by flg22 or the elongation factor-derived peptide elf18, indicating that these immune receptors likely act downstream or independently of these early PTI signaling events [79]. In the present work, we showed that another subclade of CNLs, which are targeted by miR472 and *RDR6*-dependent siRNAs, possibly contribute to multiple PTI signaling events, including potentially flg22-triggered callose deposition and ROS production (**Figure 3B**). Interestingly, the product of one if this mRNA target, the RPS5 protein, was previously shown to reside in the same protein complex as the PTI receptor FLS2 [80], further supporting a molecular link between ETI and PTI components.

### Conclusion

In conclusion we have established a direct link between *miR472*/*RDR6*-dependent PTGS and plant immunity. We showed that both *miR472* and *RDR6* act as negative regulators of PTI and ETI, presumably by repressing a subset of CNLs at the post-transcriptional level. Our data therefore sustain previous anticipations suggesting that in addition to their role in specific resistance, R proteins contribute to PTI [10], [64], [78], [81], [82]. Furthermore, given that flg22 as well as disarmed bacteria were shown to trigger Systemic Acquired Resistance (SAR), such as in response to pathogens expressing Avr products [83]–[86], we speculate that a potential release of *miR472*- and eventually of *RDR6*-dependent PTGS may also occur in distal tissues, and thereby might contribute to the transient derepression of a whole repertoire of disease resistance genes as part of the SAR response.

## Materials and Methods

### Plants and bacterial strains

*Arabidopsis thaliana* seeds from the Col-0 accession were used as wild-type, the *rdr6-15* T-DNA insertion line has been previously described in Xi et al [87]. We also used *sid2-2*, *npr1-1* and *rar1-21* mutant alleles. Plants were genotyped with the following primers and conditions: *rdr6* (RDR6_LP:TGAATCCATTCCTGAACAAGC; RDR6_RP: CAATGCAACCTCATCTTGGATG; LB3: TAGCATCTGAATTTCATAACCAATCTCGATACAC), *npr1*(1g64280_F: AGGGGATATACGGTGCTTCAT; 1g64280_R: GAGCAGCGTCATCTTCAATTC); sid2 (sid2_F:CAGTCCGAAAGACGACCTCGAGTT;sid2_R:CTCATCATCTTCCTTCGTAAGTCTCC); rar1 (5g51700_F: AAGCAGGGAGTAAGTCAAATTTAC; 5g51700_R CAAACTGAAATCATGACTTCTTTG). All plants were grown in short days conditions subjected to a cycle of 8 h and 16 h of light and darkness, respectively, at a day/night temperature of 22.5/18.5° with 50–60% humidity for about 5–6 weeks. The plants were watered 16 h before inoculation to promote stomatal opening, thereby facilitating inoculation.

*Pseudomonas syringae* pv. *tomato* DC3000 (*Pto* DC3000) was grown at 28°C on NYGB medium (5 g L^−1^ bactopeptone, 3 g L^−1^ yeast extract, 20 ml L^−1^ glycerol) containing kanamycin (50 mg mL^−1^) and rifampicin (25 mg mL^−1^) for selection.

### Plant inoculations and bacterial counting

*Pto* DC3000, *Pto* DC3000 AvrPphB and *Pto* DC3000 AvrRpt2 from overnight culture were collected, washed once and resuspended in 10 mM MgCl_2_ at a concentration of 5×10^5^ colony-forming units (CFU) mL^−1^. *A. thaliana* leaves were infiltrated with bacterial suspensions using a needleless syringe. Leaves were harvested immediately (0 dpi) or after 4 days. Two leaf discs (d = 0.4 mm) from two different leaves were washed in 10 mM MgCl_2_ and then ground with a Microfuge pestle. After grinding of the tissue, the samples were diluted 1∶10 serially. Samples were plated on NYGA solid medium (NYGB with 10 g L^−1^ agar) supplemented with antibiotics. Plates were placed at 28°C for 4 days and the CFU were counted. For spray inoculation bacteria were resuspended in 10 mM MgCl_2_ at OD_600_ of 0.2 (10^8^ CFU/mL) and Silwet was added to a final concentration of 0.04%. All experiments presented were repeated three times and statistical differences were detected with a Wilcoxon test (*, P<0.05; **, P<0.01).

### ROS measurements and callose staining

Reactive oxygen species released by leaf discs were assayed by H_2_O_2_-dependent luminescence of luminal [88]. Leaf discs were deposed into 96-well plate and incubated overnight in 200 µL H_2_O in a growth chamber. The next morning, 100 µL H_2_O containing 20 µM luminol and 1 µg horseradish peroxidase (Sigma) with or without 100 nM flg22 were added. Luminescence was immediately measured for 45 min using a Tristar LB 941 plate reader (Berthold technologies, Thoiry). At least 25 to 30 discs were tested by conditions.

For callose detection, leaves were infiltrated with 100 nM flg22 or water using a needleless syringe. After 15 h, about ten leaves from at least four independent plants were cleared by immersion in an alcoholic lactophenol solution by the method of Shipton and Brown [89] modified by Adam and Sommerville [90]. They were rinsed in 50% ethanol, then in water. Callose was detected by staining for 30 min in 150 mM K_2_HPO_4_ (pH 9.5) buffer containing 0.01% aniline blue (Sigma-Aldrich). After staining each leaf was mounted in 50% glycerol and examined with an Olympus Macro Zoom System Microscope MVX10 fluorescent microscope (excitation filter 365 nm and barrier filter 420 nm). Representative pictures are shown. The number of callose deposits per picture was determined using ImageJ (National Institutes of Health, Bethesda, MD, U.S.A.) and compared using a Wilcoxon test (P<0.05). We analyzed 25 to 30 pictures corresponding to more than five independent leaves for each treatment.

### RNA extraction and RT-qPCR analyses

For RNA extraction, leaves or seedlings were collected, immediately frozen in liquid nitrogen, and then stored at −80°C. Total RNA was prepared by TRIzol (Invitrogen) extraction as recommended by the supplier (Invitrogen). For RT-PCR analysis, first-strand cDNA was synthesized using Superscript reverse transcriptase (Invitrogen,) from 1 µg of RNase-free DNaseI-treated (Promega) total RNA in a 20 µl reaction volume. Quantitative PCR reactions were performed on 1/40 of cDNA, 300 nM final concentration of each primer pair and LightCycler 480 SYBR Green I Master 2× conc. (Roche). PCR was performed in 384-well optical reaction plates heated at 95°C for 10 min, followed by 45 cycles of denaturation at 95°C for 15 s and annealing and elongation at 60°C for 30 s. A melting curve was performed at the end of the amplification by steps of 1°C (from 95°C to 50°C). Each experiment was repeated two to three times. Transcript levels were normalized to that of At2G36060, At4G29130 and At5G13440 genes. These reference genes display invariant expression over hundreds of publicly available microarray experiments. The gene-specific primers used in this analysis were listed in **Figure S14**.

For miR472 quantification, total RNA was isolated from plants using TRIzol reagent (Invitrogen) and treated with RNase-free DNaseI (Promega). Small RNAs were polyadenylated with ATP by poly(A) polymerase following the manufacturer's directions for the Poly(A) Tailing Kit (Ambion). After phenol-chloroform extraction and ethanol precipitation, the RNAs were reverse-transcribed with 200 U SuperScript III Reverse Transcriptase (Invitrogen) and 0.5 µg poly(T) adapter (**Figure S14**) according to the manufacturer's protocols (Invitrogen). The cDNAs were used for qPCR with miR472 as one primer and the reverse primer as described by Shi and Chiang [91]. 5,8S ribosomal RNA gene was used as internal control as previously described [91]. Sequences of miR472, reverse primer, poly(T) adapter and 5S primers are listed in **Figure S14**.

### Deep-sequencing

Total cellular RNA (5 µg), extracted using TRIzol reagent (Invitrogen) was processed into sequencing libraries using adapted Illumina protocols and sequenced at Fasteris (http://www.fasteris.com, Switzerland) using the Hi-seq 2000 sequencer. All next-generation sequencing data have been deposited to the NCBI Gene Expression Omnibus (GEO).

### Bioinformatic analyses

We took advantage of publicly available sRNA libraries from leaf tissue [92]. These data correspond to 2 replicates of WT and *rdr6* sRNA sequenced using Illumina Genome Analyser technology. Replicates were pooled and sequence reads were matched against the *Arabidopsis thaliana* genome (TAIR10) using MUMmer v3.0 [93]. Only 15 to 30-nt long sRNAs reads with perfect match over their entire length were analysed further (2 434 780 and 1 753 064 for WT and *rdr6* respectively). The number of 20–22 nt reads matching TAIR10 annotated protein coding genes locus or tasiRNA were then compared between WT and *rdr6* libraries by differential analysis with NOISeq [94] using the parameters indicated below: k = NULL, norm = “rpkm”, long = 1000, q = 0.90, pnr = 0.5, nss = 1000, v = 0.02, lc = 1.

The WT and miR472OE sRNA libraries, containing 17 828 872 and 30 869 878 sRNA reads respectively, were processed using the same methods. Over those reads, 88.9% are 15 to 30-nt long and can be perfectly aligned to Arabidopsis genome. The miR472OE line was validated by comparing the number of reads mapping to all miRNA stem-loop loci (miRBase release 19; [95]–[98]) between WT and mutant sRNA libraries. Differential analysis of 20–22 nt reads in genes has then been done as described in the previous paragraph.

## Supporting Information

Figure S1 **Transcript levels of several PTGS genes in response to biotic stresses.** (**A**) The results of 48 different conditions of perturbation (Genevestigator: https://www.genevestigator.com) were compiled and boxplots were generated for each PTGS gene. Relative expression is the ratio (Log_2_) between treated and untreated plants. (**B**) Relative RDR6 mRNA levels in plants treated with PAMPs, microbial elicitors or *P. syringae* non-host (data from Genevestigator). (**C**) Transcript levels of RDR6 and AGO1 detected by RT-qPCR. (Left) seedling treated with flagellin for 10, 30 and 45 min. (Right) Plants infiltrated with *Pto* DC3000 *hrcC* ^−^ or *Pto* DC3000. Expression levels are relative to three reference genes (At2g36060; At4g29130; At5g13440). The Log_2_ of mRNA values is normalized to that of WT plants treated with water (seedlings) or infiltrated with MgCl_2_ (leaves). Error bars indicate standard deviation from technical repeats. Similar results were obtained in two biological replicates.
(TIF)

Figure S2 **List of genes from WT and** ***rdr6*** **sRNAs public libraries producing** ***RDR6*** **-dependent siRNAs, which are not** ***TAS*** **genes, nor tasiRNAs targets.**
(PDF)

Figure S3 **Genes targeted by microRNAs and** ***RDR6*** **-dependent siRNAs.**
(PDF)

Figure S4 **Snap shot of two resistance genes, At1g51480 (RSG1) and At5g43730 (RSG2), showing a reduced number of 21–22 nt siRNAs in** ***rdr6*** **background compared to WT leaves.**
(TIF)

Figure S5 **CNL transcripts accumulate after flg22 treatment.** Ratio (Log_2_) of At1g12220 (RPS5), At1g51480 (RSG1) and At5g43730 (RSG2) expression levels between flg22-treated and untreated leaf discs with flg22 for 1 hour and 2 hours from publicly available microarrays data (Genevestigator: https://www.genevestigator.com).
(TIF)

Figure S6 **Expression levels of At1g12220 and At1g51480 detected by RT-qPCR in WT, and miR472OE (overexpressor) untreated seedlings.**
(TIF)

Figure S7 **List of genes, which accumulate more siRNAs (21–22 nt) in miR472OE than in WT.** In bold: resistance genes, in italic: putative targets of secondary siRNAs.
(PDF)

Figure S8 **Mature Tasi RNAs accumulation is not affected in miR472OE line.**
(TIF)

Figure S9 **Snap shot of two resistance genes At5g43730 (RSG2) and At1g12280 (SUMM2), which accumulate 21–22 nt siRNAs in miR472OE.**
(TIF)

Figure S10 **Transgenic plants overexpressing miR472 show reduced PTI responses and are more susceptible to** ***Pto*** **DC3000.** (**A**) Callose deposition induced by flg22 (100 nM) in WT, miR472OE 1 and 2 lines (**B**) Bacterial growth in five- to six-week-old plants from WT, miR472OE 1 and 2 lines were infiltrated with *Pto* DC3000 (2 10^5^ CFU mL^−1^). Values are average ± se of four leaf discs (n = 8). Wilcoxon test was performed to determine the significant differences as compared to WT plants. Asterisks “*” and “**” indicate statistically significant differences at a P value<0.05 and <0.01 respectively.
(TIF)

Figure S11 **MiR472 accumulation in WT,** ***rdr6*** **and** ***miR472^m^*** **plants.** Relative microRNAs accumulation was measured by RT-qPCR as described in [91]. MiR163, which exhibits an experimental Tm similar to that of miR472 was used as control.
(TIF)

Figure S12 **Resistance to Pto DC3000 AvrRpt2 is not affected in** ***MiR472*** **^m^ and** ***rdr6*** **mutants.** Bacterial growth in WT, *rdr6* and *miR472^m^* plants infiltrated with *Pto* DC3000 AvrRPT2 (2 10^5^ CFU mL^−1^).
(TIF)

Figure S13 **MiR472 accumulation in WT seedlings or leaves treated with flagellin.** Seedlings were treated with water or 100 nM flg22 for 30 min, 1, 2 and 4 hours and leaves infiltrated for 3, 6 and 9 hours with water or 100 nM flg22. Relative microRNAs accumulation was measured by RT-qPCR as described in [91]. MiR163, which exhibits an experimental Tm similar to that of miR472, was used as control. Error bars indicate standard deviation from technical repeats. Similar results were obtained in two independent experiments.
(TIF)

Figure S14 **List of primers used in this study.**
(PDF)
