# Correction: The Arabidopsis *miR472-RDR6* Silencing Pathway Modulates PAMP- and Effector-Triggered Immunity through the Post-transcriptional Control of Disease Resistance Genes

**DOI:** 10.1371/journal.ppat.1004814

**Published:** 2015-04-10

**Authors:** Martine Boccara, Alexis Sarazin, Odon Thiébeauld, Florence Jay, Olivier Voinnet, Lionel Navarro, Vincent Colot

S7 Figure in the article is a duplicate of S14 Figure. There was an error in the deposition of the S7 Fig. file, please see the correct version of S7 Figure here. The Supporting Information legends for these figures are unchanged.

## Supporting Information

S7 Fig List of genes, which accumulate more siRNAs (21–22 nt) in miR472OE than in WT. In bold: resistance genes, in italic: putative targets of secondary siRNAs.
(PDF)
