# Supplementary figures and images for: The Arabidopsis miR472-RDR6 Silencing Pathway Modulates PAMP- and Effector-Triggered Immunity through the Post-transcriptional Control of Disease Resistance Genes
Source: PLoS Pathog. 2014 Jan 16;10(1):e1003883. doi: 10.1371/journal.ppat.1003883 (PMC3894208; doi:10.1371/journal.ppat.1003883)

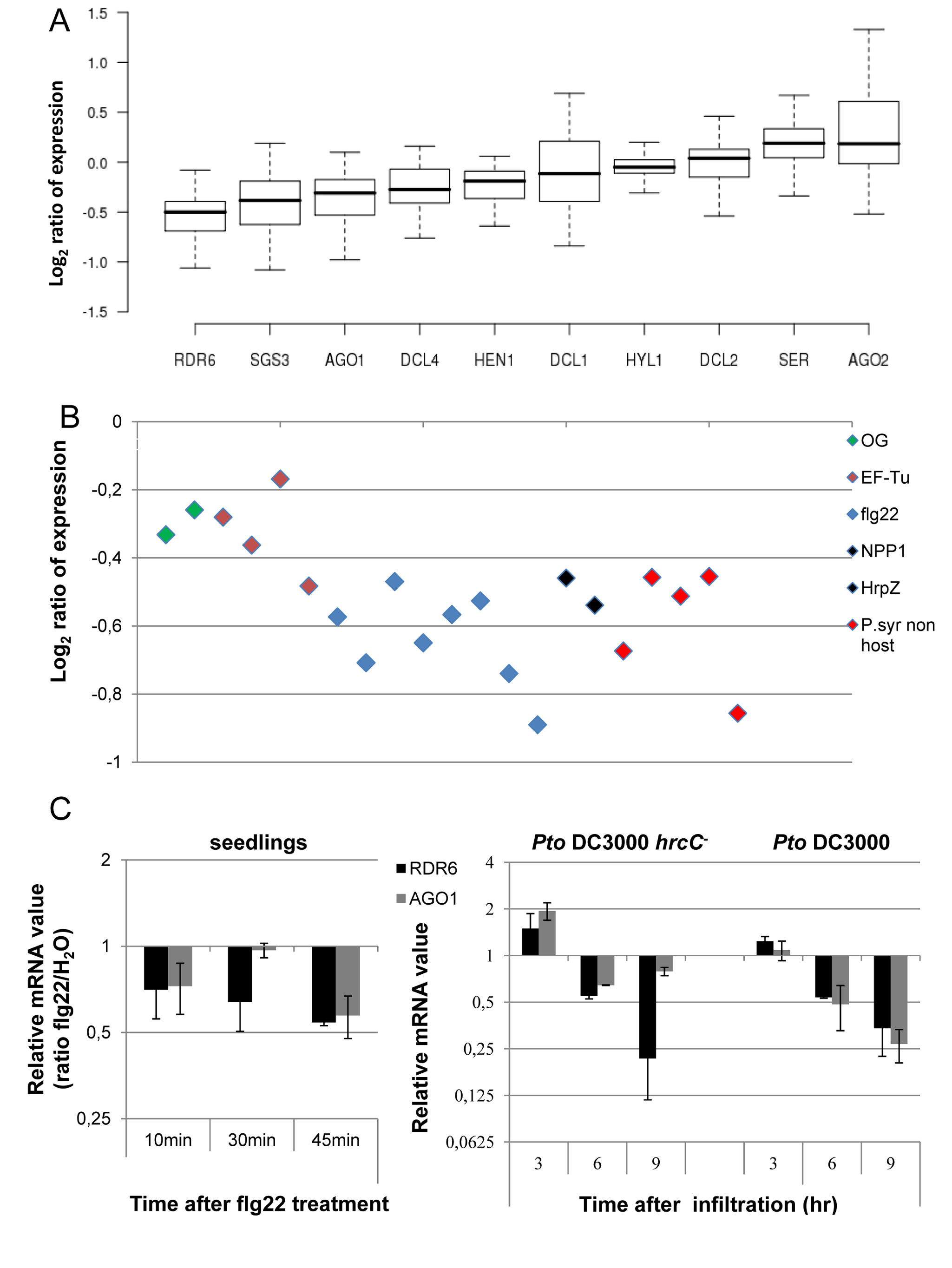

Supplement: Figure S1 — Transcript levels of several PTGS genes in response to biotic stresses. (A) The results of 48 different conditions of perturbation (Genevestigator: https://www.genevestigator.com) were compiled and boxplots were generated for each PTGS gene. Relative expression is the ratio (Log2) between treated and untreated plants. (B) Relative RDR6 mRNA levels in plants treated with PAMPs, microbial elicitors or P. syringae non-host (data from Genevestigator). (C) Transcript levels of RDR6 and AGO1 detected by RT-qPCR. (Left) seedling treated with flagellin for 10, 30 and 45 min. (Right) Plants infiltrated with Pto DC3000 hrcC − or Pto DC3000. Expression levels are relative to three reference genes (At2g36060; At4g29130; At5g13440). The Log2 of mRNA values is normalized to that of WT plants treated with water (seedlings) or infiltrated with MgCl2 (leaves). Error bars indicate standard deviation from technical repeats. Similar results were obtained in two biological replicates. (TIF) [file ppat.1003883.s001.tif]

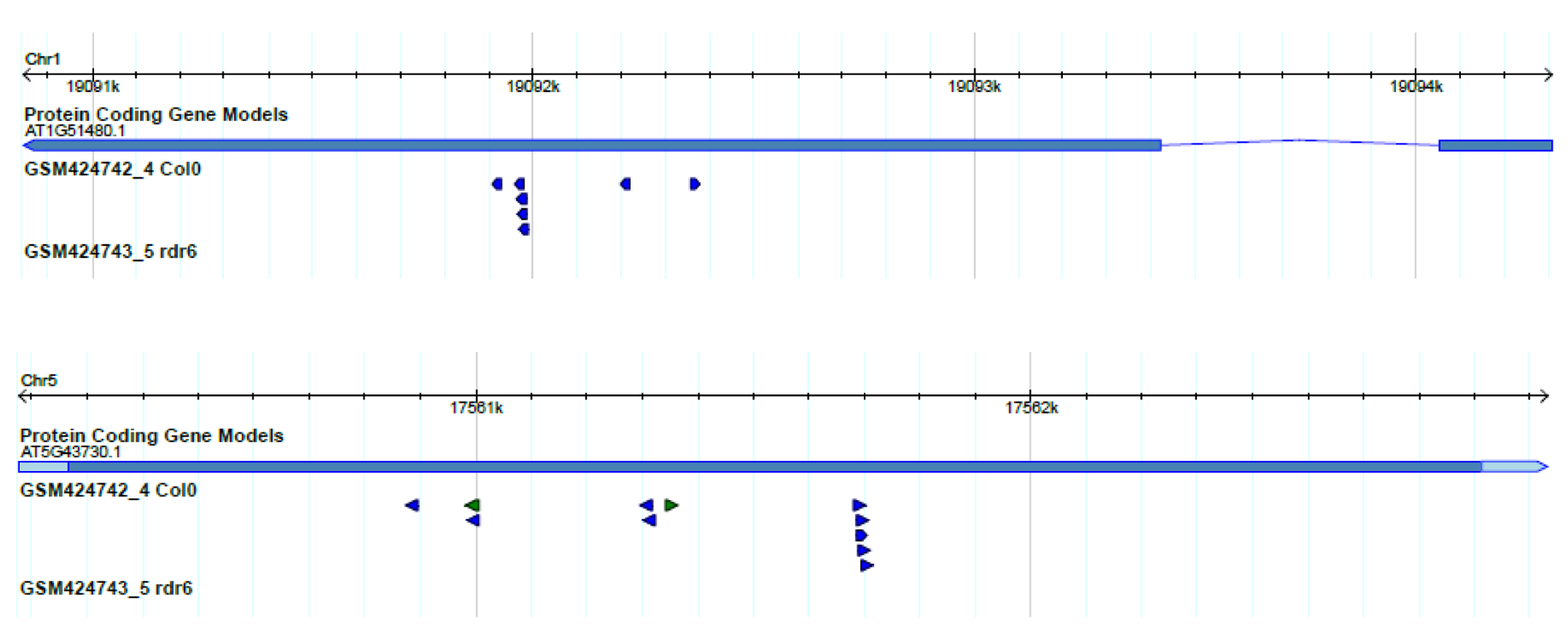

Supplement: Figure S4 — Snap shot of two resistance genes, At1g51480 (RSG1) and At5g43730 (RSG2), showing a reduced number of 21–22 nt siRNAs in rdr6 background compared to WT leaves. (TIF) [file ppat.1003883.s004.tif]

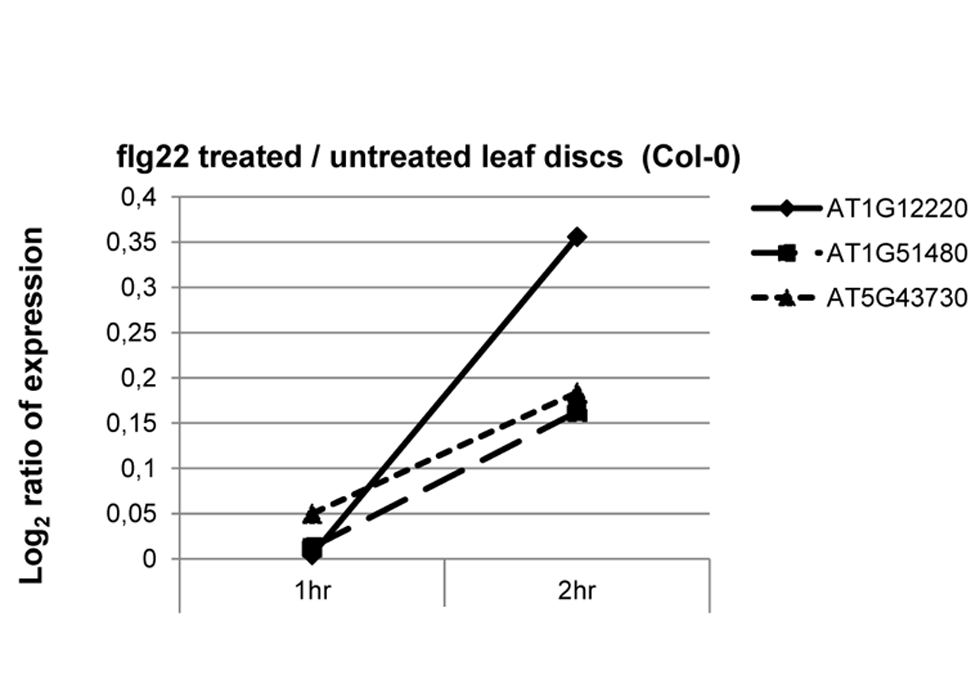

Supplement: Figure S5 — CNL transcripts accumulate after flg22 treatment. Ratio (Log2) of At1g12220 (RPS5), At1g51480 (RSG1) and At5g43730 (RSG2) expression levels between flg22-treated and untreated leaf discs with flg22 for 1 hour and 2 hours from publicly available microarrays data (Genevestigator: https://www.genevestigator.com). (TIF) [file ppat.1003883.s005.tif]

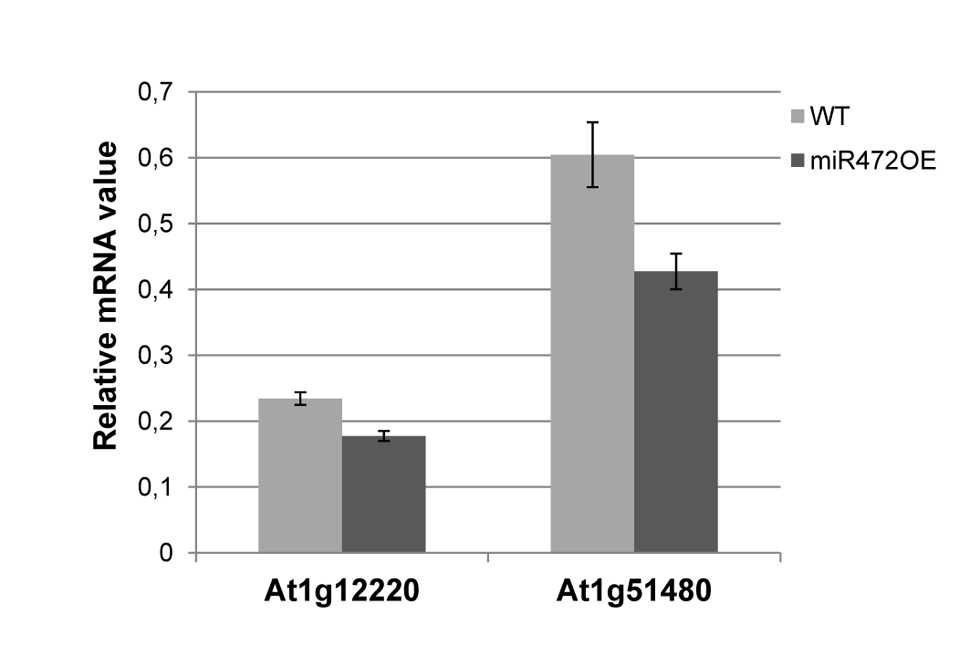

Supplement: Figure S6 — Expression levels of At1g12220 and At1g51480 detected by RT-qPCR in WT, and miR472OE (overexpressor) untreated seedlings. (TIF) [file ppat.1003883.s006.tif]

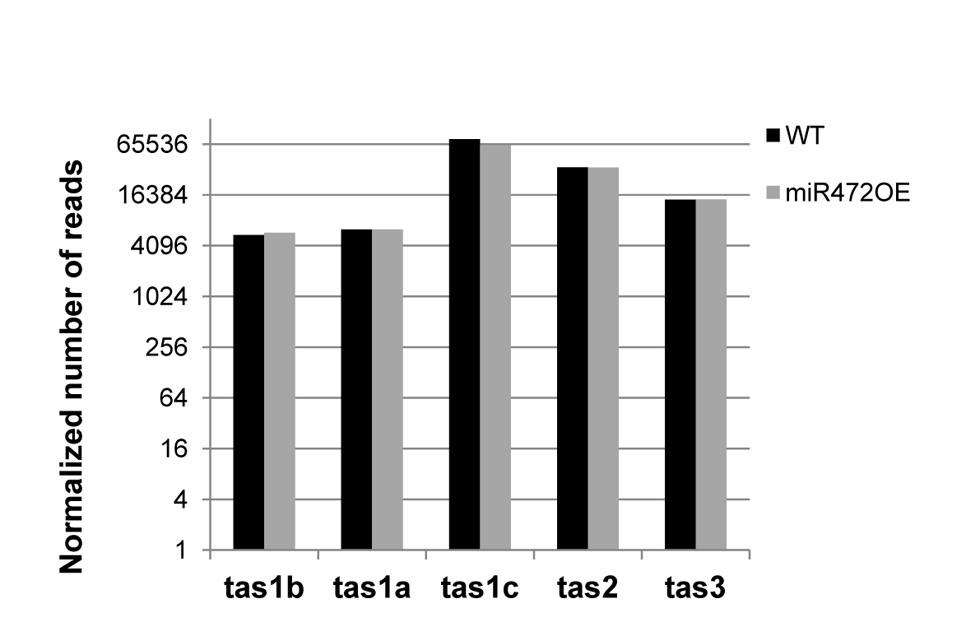

Supplement: Figure S8 — Mature Tasi RNAs accumulation is not affected in miR472OE line. (TIF) [file ppat.1003883.s008.tif]

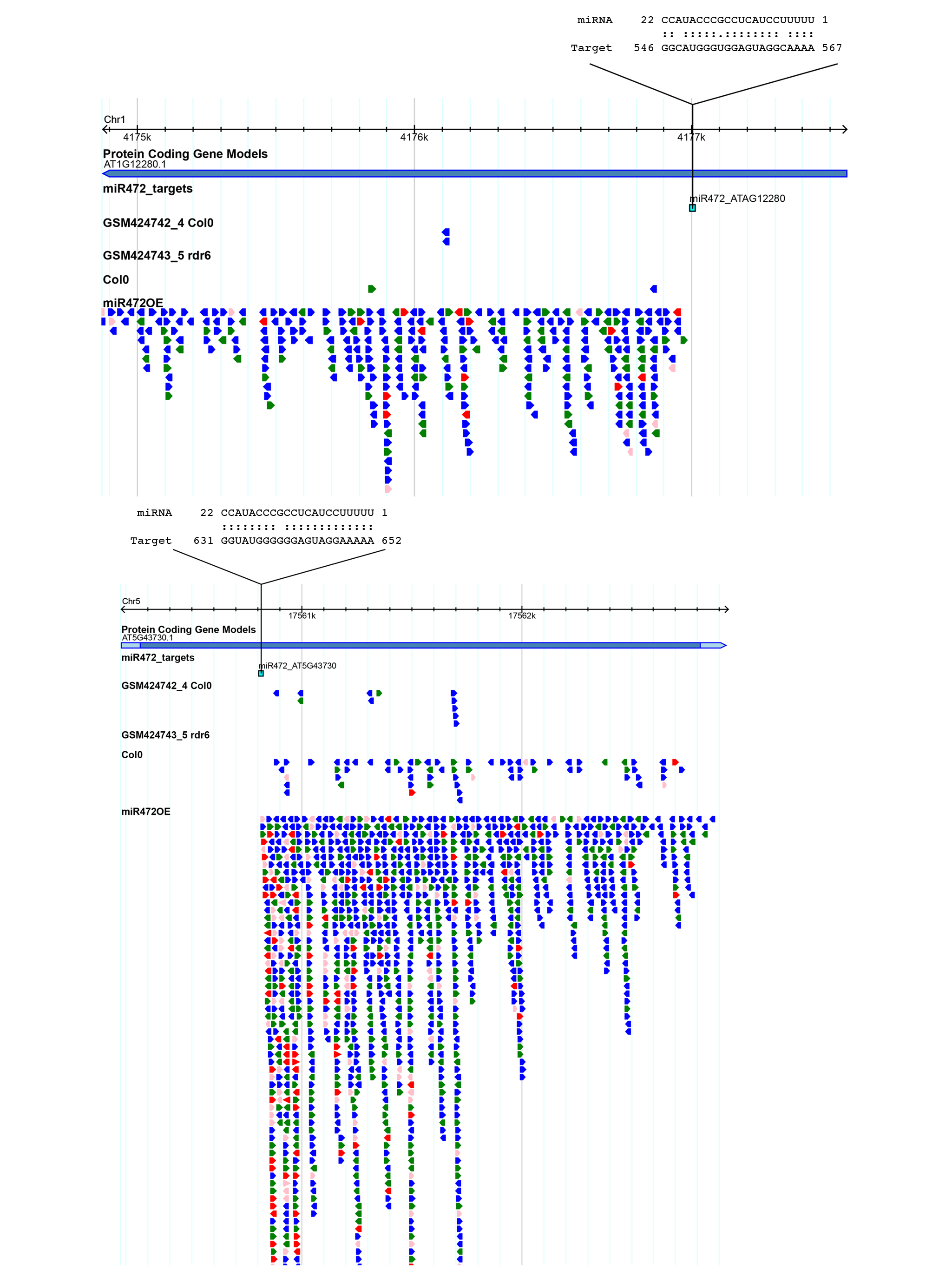

Supplement: Figure S9 — Snap shot of two resistance genes At5g43730 (RSG2) and At1g12280 (SUMM2), which accumulate 21–22 nt siRNAs in miR472OE. (TIF) [file ppat.1003883.s009.tif]

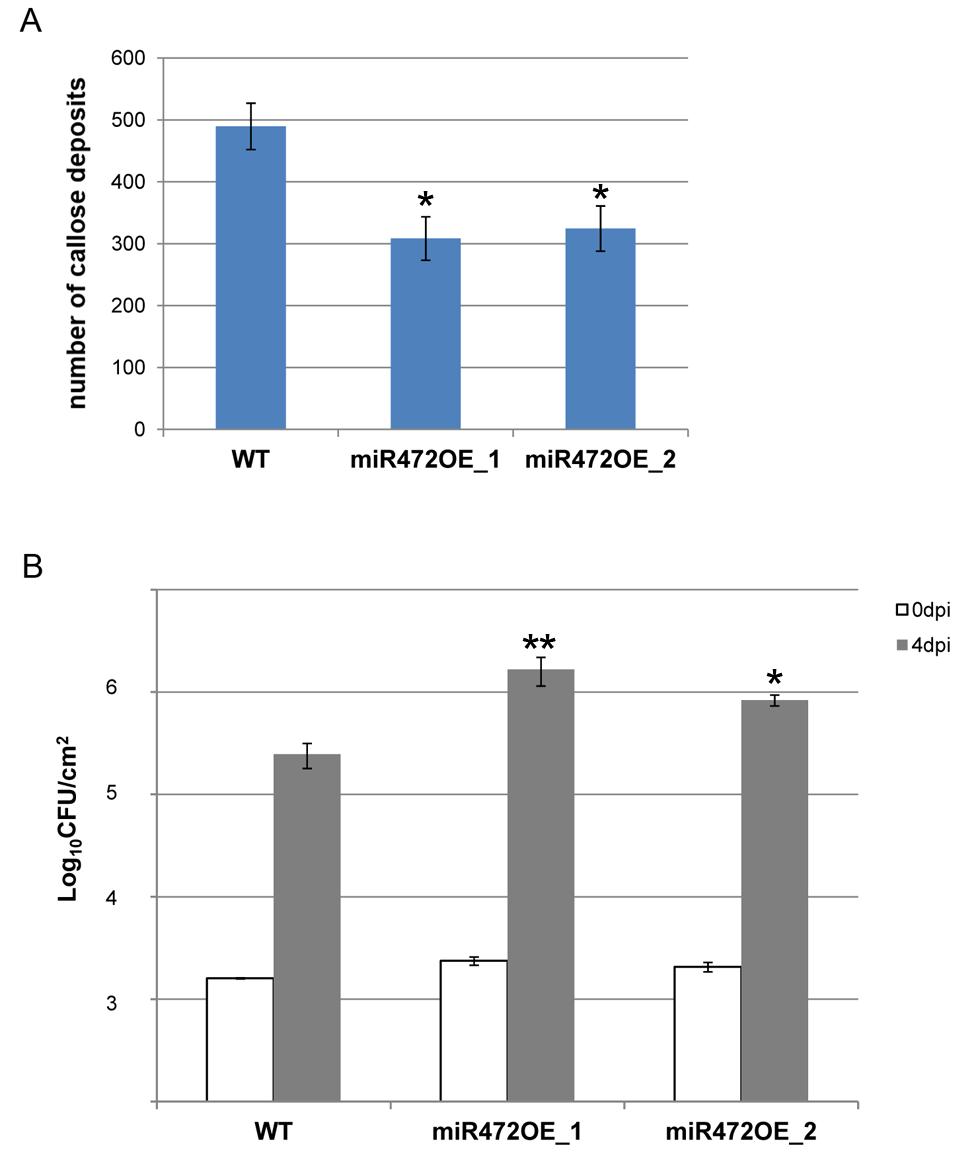

Supplement: Figure S10 — Transgenic plants overexpressing miR472 show reduced PTI responses and are more susceptible to Pto DC3000. (A) Callose deposition induced by flg22 (100 nM) in WT, miR472OE 1 and 2 lines (B) Bacterial growth in five- to six-week-old plants from WT, miR472OE 1 and 2 lines were infiltrated with Pto DC3000 (2 105 CFU mL−1). Values are average ± se of four leaf discs (n = 8). Wilcoxon test was performed to determine the significant differences as compared to WT plants. Asterisks “*” and “**” indicate statistically significant differences at a P value<0.05 and <0.01 respectively. (TIF) [file ppat.1003883.s010.tif]

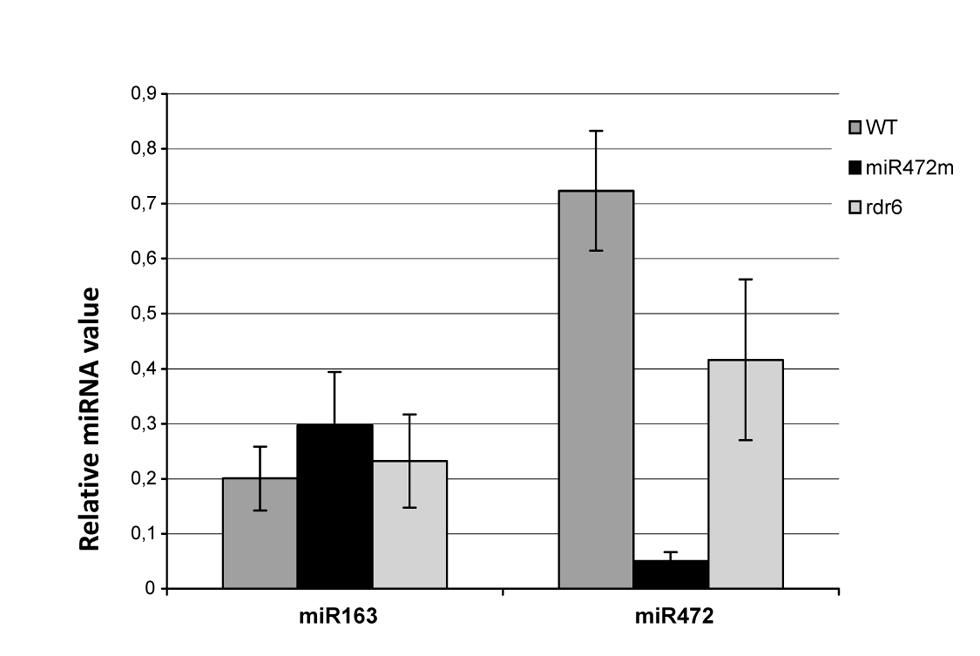

Supplement: Figure S11 — MiR472 accumulation in WT, rdr6 and miR472m plants. Relative microRNAs accumulation was measured by RT-qPCR as described in [91]. MiR163, which exhibits an experimental Tm similar to that of miR472 was used as control. (TIF) [file ppat.1003883.s011.tif]

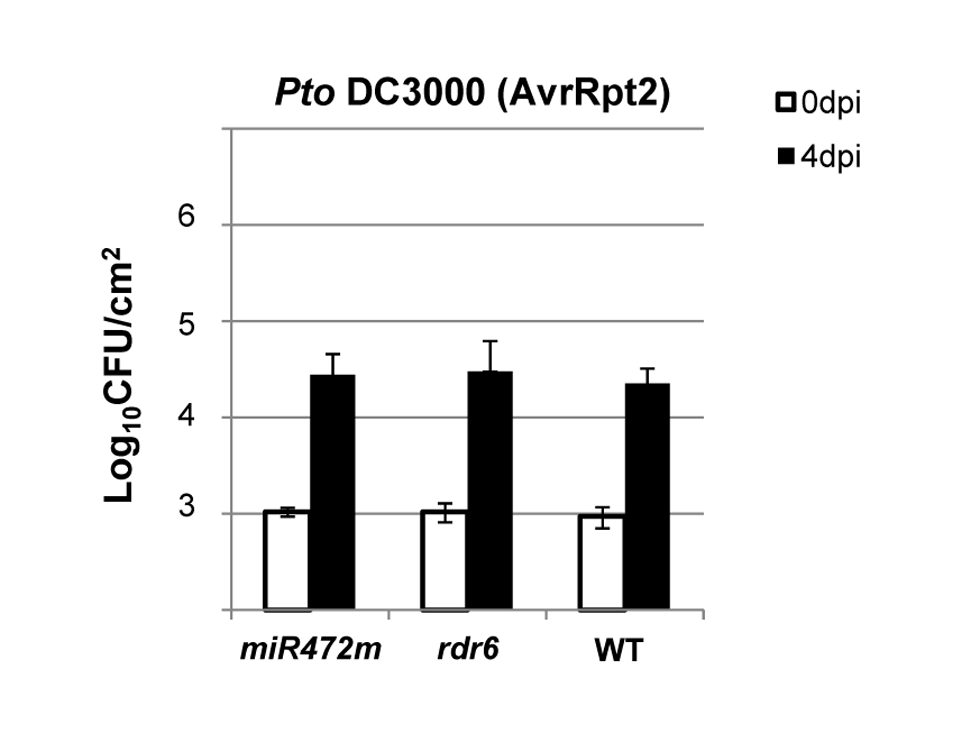

Supplement: Figure S12 — Resistance to Pto DC3000 AvrRpt2 is not affected in MiR472 m and rdr6 mutants. Bacterial growth in WT, rdr6 and miR472m plants infiltrated with Pto DC3000 AvrRPT2 (2 105 CFU mL−1). (TIF) [file ppat.1003883.s012.tif]

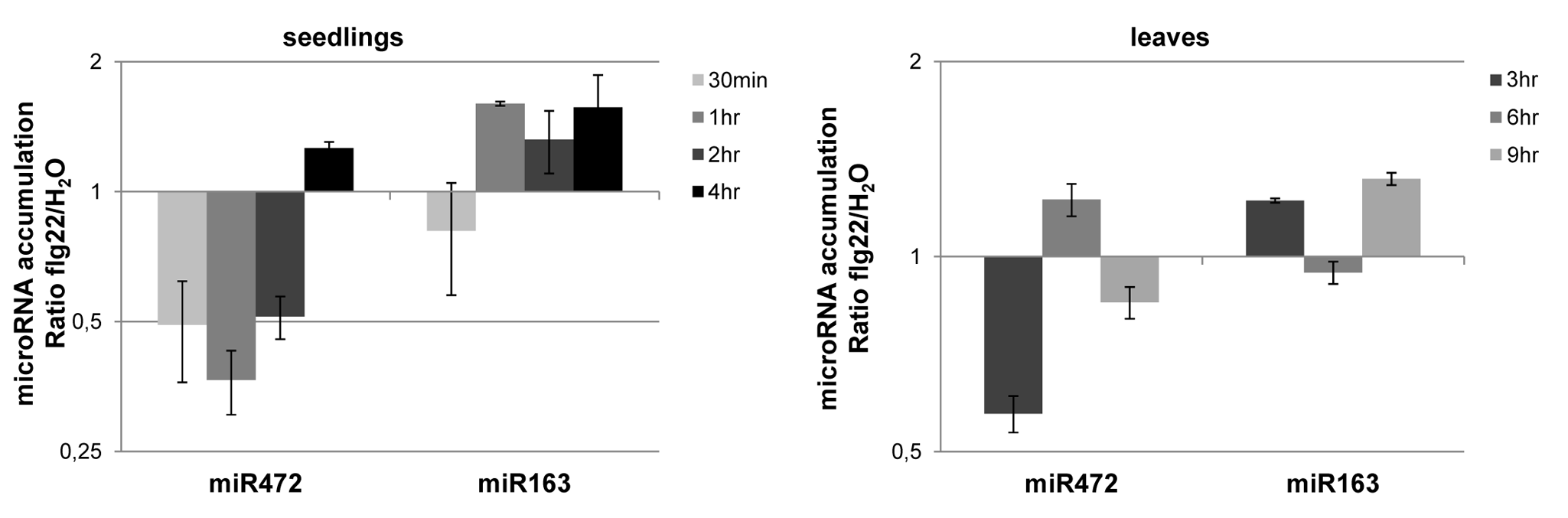

Supplement: Figure S13 — MiR472 accumulation in WT seedlings or leaves treated with flagellin. Seedlings were treated with water or 100 nM flg22 for 30 min, 1, 2 and 4 hours and leaves infiltrated for 3, 6 and 9 hours with water or 100 nM flg22. Relative microRNAs accumulation was measured by RT-qPCR as described in [91]. MiR163, which exhibits an experimental Tm similar to that of miR472, was used as control. Error bars indicate standard deviation from technical repeats. Similar results were obtained in two independent experiments. (TIF) [file ppat.1003883.s013.tif]
