# Supplementary material for: The Arabidopsis miR472-RDR6 Silencing Pathway Modulates PAMP- and Effector-Triggered Immunity through the Post-transcriptional Control of Disease Resistance Genes
Source: PLoS Pathog. 2014 Jan 16;10(1):e1003883. doi: 10.1371/journal.ppat.1003883 (PMC3894208; doi:10.1371/journal.ppat.1003883)
Supplement: Figure S2 — List of genes from WT and rdr6 sRNAs public libraries producing RDR6 -dependent siRNAs, which are not TAS genes, nor tasiRNAs targets. (PDF) [file ppat.1003883.s002.pdf]

Gene id[1]	Function
AT1G01720	NAC (No Apical Meristem) domain transcriptional regulator superfamily protein
**AT1G12820**	auxin signaling F-box 3
AT1G13360	unknown
AT1G29910	chlorophyll A/B binding protein 3
AT1G40390	DNAse I-like superfamily protein
AT1G48410	AGO1 slicer
**AT1G51480**	Disease resistance protein (CC-NBS-LRR class) family
**AT1G62630**	Disease resistance protein (CC-NBS-LRR class) family
AT1G62670	rna processing factor 2
AT1G62914	pentatricopeptide (PPR) repeat-containing protein
**AT1G63360**	Disease resistance protein (CC-NBS-LRR class) family
**AT1G63870**	Disease resistance protein (TIR-NBS-LRR class) family
**AT1G63880**	Disease resistance protein (TIR-NBS-LRR class) family
AT1G64583	Tetratricopeptide repeat (TPR)-like superfamily protein
AT1G67870	glycine-rich protein
AT1G68840	related to ABI3/VP1 2
**AT1G74100**	sulfotransferase 16
AT2G03330	unknown
AT2G20950	Arabidopsis phospholipase-like protein (PEARLI 4) family
AT2G25370	RING/U-box superfamily protein
AT2G25510	unknown
**AT3G07390**	auxin-responsive family protein
AT3G15730	phospholipase D alpha 1
**AT3G23690**	basic helix-loop-helix (bHLH) DNA-binding superfamily protein
**AT3G26810**	auxin signaling F-box 2
AT3G27400	Pectin lyase-like superfamily protein
AT3G32200	unknown
AT3G43270	Plant invertase/pectin methylesterase inhibitor superfamily
AT3G45820	unknown
**AT3G46550**	Fasciclin-like arabinogalactan family protein
AT3G57800	basic helix-loop-helix (bHLH) DNA-binding superfamily protein
**AT3G62980**	F-box/RNI-like superfamily protein [2]
AT4G04423	unknown
AT4G13340	Leucine-rich repeat (LRR) family protein
**AT4G14140**	DNA methyltransferase 2
AT4G14210	phytoene desaturase 3
AT4G22770	AT hook motif DNA-binding family protein
AT5G08130	basic helix-loop-helix (bHLH) DNA-binding superfamily protein
AT5G09670	loricrin-related
AT5G09672	conserved peptide upstream open reading frame 21
AT5G26290	TRAF-like family protein

[1] Italic: Argonaute 1
[2] Bold: stress responsive genes

AT5G27890	unknown
AT5G35604	unknown
**AT5G38850**	Disease resistance protein (TIR-NBS-LRR class)
**AT5G43730**	Disease resistance protein (CC-NBS-LRR class) family
**AT5G43740**	Disease resistance protein (CC-NBS-LRR class) family
**AT5G63020**	Disease resistance protein (CC-NBS-LRR class) family
AT5G66380	folate transporter 1
