# Supplementary material for: The Arabidopsis miR472-RDR6 Silencing Pathway Modulates PAMP- and Effector-Triggered Immunity through the Post-transcriptional Control of Disease Resistance Genes
Source: PLoS Pathog. 2014 Jan 16;10(1):e1003883. doi: 10.1371/journal.ppat.1003883 (PMC3894208; doi:10.1371/journal.ppat.1003883)
Supplement: Figure S3 — Genes targeted by microRNAs and RDR6 -dependent siRNAs. (PDF) [file ppat.1003883.s003.pdf]

microRNA	Target	Name, family	function
miR161	AT1G62670	PPR	RNA chaperone
miR168	AT1G48410	AGO1	slicer
miR393	AT1G12820<br>AT3G26810<br>AT3G23690<br>AT3G62980	AFB3<br>AFB2<br>ARF3<br>TIR1	Innate immunity
miR400a	AT1G62670	RPF2 (PPR)	RNA processing factor
miR472	AT1G51480<br>AT1G62630<br>AT1G63360<br>AT5G63020<br>AT5G43730<br>AT5G43740	R proteins CC-NBS-LRR	Resistance to pathogens
miR773a	AT4G14140	Met2	DNA methyltransferase?
