# Supplementary material for: The Arabidopsis miR472-RDR6 Silencing Pathway Modulates PAMP- and Effector-Triggered Immunity through the Post-transcriptional Control of Disease Resistance Genes
Source: PLoS Pathog. 2014 Jan 16;10(1):e1003883. doi: 10.1371/journal.ppat.1003883 (PMC3894208; doi:10.1371/journal.ppat.1003883)
Supplement: Figure S7 — List of genes, which accumulate more siRNAs (21–22 nt) in miR472OE than in WT. In bold: resistance genes, in italic: putative targets of secondary siRNAs. (PDF) [file ppat.1003883.s007.pdf]

name	sequence	comments
At1g12220_5pF	TCTTTGTGGTTTCTGCTCCA	CC-NB-LRR
At1g12220_5pR	GGATCTCATCCACATCAGCA	
At1g51480_5pF	CTTGTCCGTGCATGTTTGTT	CC-NB-LRR
At1g51480_5pR	ACGTAACCCAACACCAGCTC	
At5g43730_5pF	GAATGGGCGAGATGATCTGT	CC-NB-LRR
At5g43730_5pR	CCAGTTTCGATTGACATAGCC	
PDF1.2 F	CACCCTTATCTTCGCTGCTC	Marker of JA and SA pathway
PDF1.2 R	GTTGCATGATCCATGTTTGG	
PR1_F	TCGTCTTTGTAGCTCTTGTAGGTG	Marker of SA pathway
PR1_R	TAGATTCTCGTAATCTCAGCTCT	
RDR6_F	TGCAATCCAAGCAAAACTCA	
RDR6_R	TGAGGAAACAATCCCTGACC	
AGO1_F	AGAGAAGAACGGATGCTCCA	
AGO1_R	CACCTTGGTGTTGTCCTCCT	
WRK22_F	TCCTTCGGAGAGATTCGAGA	PTI marker
WRK22_R	CTGCTGCTACATGGCACACT	
WRK29_F	CCCGGAGAAATTCACCATAA	PTI marker
WRK29_R	ATCAGCGGATGGGATCATAG	
FRK1_F	TATCTTGAGCTGGGAAGAGAGG	PTI marker
FRK1_R	AGTCGAATAGTACTCGGGGTCA	
poly(T) adapter	GCGAGCACAGAATTAATACGACTCACTATAG G(T)12VN	
Reverse primer	GCGAGCACAGAATTAATACGAC	
miR472	TTTTTCCTACTCCGCCCATACC	
AT5G13440F	ACAAGCCAATTTTTGCTGAGC	Reference gene for qPCR
AT5G13440R	ACAACAGTCCGAGTGTCATGGT	
AT2G36060F	TGAAGTCGTGAGACAGCGTTG	Reference gene for qPCR
AT2G36060R	GGGCTTCTCCATTGTTGGTC	
AT4G29130F	GGCGTTTTCTGATAGCGAAAA	Reference gene for qPCR
AT4G29130R	ATGGATCAGGCATTGGAGCT	
