# Supplementary material for: The Arabidopsis miR472-RDR6 Silencing Pathway Modulates PAMP- and Effector-Triggered Immunity through the Post-transcriptional Control of Disease Resistance Genes
Source: PLoS Pathog. 2014 Jan 16;10(1):e1003883. doi: 10.1371/journal.ppat.1003883 (PMC3894208; doi:10.1371/journal.ppat.1003883)
Supplement: Figure S14 — List of primers used in this study. (PDF) [file ppat.1003883.s014.pdf]

name	sequence	comments
At1g12220_5pF	TCTTTGTGGTTTCTGCTCCA	CC-NB-LRR
At1g12220_5pR	GGATCTCATCCACATCAGCA	
At1g51480_5pF	CTTGTCCGTGCATGTTTGTT	CC-NB-LRR
At1g51480_5pR	ACGTAACCCAACACCAGCTC	
At5g43730_5pF	GAATGGGCGAGATGATCTGT	CC-NB-LRR
At5g43730_5pR	CCAGTTTCGATTGACATAGCC	
ICS1_F	CCTAATGGCAAGATCGCTGT	Biosynthesis of salicylic acid (SA)
ICS1_R	AGCAATAGTTGCAGCCAACA	
PR1_F	TCGTCTTTGTAGCTCTTGTAGGTG	Marker of SA pathway
PR1_R	TAGATTCTCGTAATCTCAGCTCT	
RDR6_F	TGCAATCCAAGCAAAACTCA	
RDR6_R	TGAGGAAACAATCCCTGACC	
AGO1_F	AGAGAAGAACGGATGCTCCA	
AGO1_R	CACCTTGGTGTTGTCCTCCT	
WRK22_F	TCCTTCGGAGAGATTCGAGA	PTI marker
WRK22_R	CTGCTGCTACATGGCACACT	
WRK29_F	CCCGGAGAAATTCACCATAA	PTI marker
WRK29_R	ATCAGCGGATGGGATCATAG	
FRK1_F	TATCTTGAGCTGGGAAGAGAGG	PTI marker
FRK1_R	AGTCGAATAGTACTCGGGGTCA	
poly(T) adapter	GCGAGCACAGAATTAATACGACTCACTATAGG(T)12VN	
Reverse primer	GCGAGCACAGAATTAATACGAC	
miR472	TTTTTCCTACTCCGCCCATACC	
AT5G13440F	ACAAGCCAATTTTTGCTGAGC	Refernce for qPCR
AT5G13440R	ACAACAGTCCGAGTGTCATGGT	
AT4G29130F	GGCGTTTTCTGATAGCGAAAA	Refernce for qPCR
AT4G29130R	ATGGATCAGGCATTGGAGCT	
