# Supplementary material for: Correction: The Arabidopsis miR472-RDR6 Silencing Pathway Modulates PAMP- and Effector-Triggered Immunity through the Post-transcriptional Control of Disease Resistance Genes
Source: PLoS Pathog. 2015 Apr 10;11(4):e1004814. doi: 10.1371/journal.ppat.1004814 (PMC4393137; doi:10.1371/journal.ppat.1004814)
Supplement: S7 Fig — In bold: resistance genes, in italic: putative targets of secondary siRNAs. (PDF) [file ppat.1004814.s001.pdf]

AT1G06760	*winged-helix DNA-binding transcription factor family protein*
**AT1G12210**	**RPS5-like 1**
**AT1G12220**	**Disease resistance protein (CC-NBS-LRR class) family (RPS5)**
**AT1G12280**	**LRR and NB-ARC domains-containing disease resistance protein**
**AT1G12290**	**Disease resistance protein (CC-NBS-LRR class) family**
**AT1G15890**	**Disease resistance protein (CC-NBS-LRR class) family**
**AT1G51480**	**Disease resistance protein (CC-NBS-LRR class) family**
**AT1G53350**	**Disease resistance protein (CC-NBS-LRR class) family**
AT1G56580	*Protein of unknown function, DUF538*
AT1G59760	*RNA helicase, ATP-dependent, SK12/DOB1 protein*
**AT1G61190**	**LRR and NB-ARC domains-containing disease resistance protein**
**AT1G61300**	**LRR and NB-ARC domains-containing disease resistance protein**
**AT1G61310**	**LRR and NB-ARC domains-containing disease resistance protein**
**AT1G61180**	**LRR and NB-ARC domains-containing disease resistance protein**
**AT1G62630**	**Disease resistance protein (CC-NBS-LRR class) family**
**AT1G63350**	**Disease resistance protein (CC-NBS-LRR class) family**
**AT1G63360**	**Disease resistance protein (CC-NBS-LRR class) family**
AT2G05410	TRAF-like family protein
AT2G10602	unknown
AT2G42620	*RNI-like superfamily protein*
AT2G46505	*succinate dehydrogenase subunit 4*
AT2G46840	DOMAIN OF UNKNOWN FUNCTION 724 4
AT3G03960	*TCP-1/cpn60 chaperonin family protein*
AT3G12500	*basic chitinase*
AT3G13674	*unknown*
AT3G25840	Protein kinase superfamily protein
AT3G44510	alpha/beta-Hydrolases superfamily protein
AT3G52730	*ubiquinol-cytochrome C reductase UQCRX/QCR9-like family protein*
**AT4G10780**	**LRR and NB-ARC domains-containing disease resistance protein**
AT4G24780	*Pectin lyase-like superfamily protein*
AT5G11370	FBD / Leucine Rich Repeat domains containing protein
AT5G18580	*tonneau 2 (TON2)*
AT5G21125	unknown
AT5G40020	Pathogenesis-related thaumatin superfamily protein
**AT5G43730**	**Disease resistance protein (CC-NBS-LRR class) family**
**AT5G43740**	**Disease resistance protein (CC-NBS-LRR class) family**
**AT5G47250**	**LRR and NB-ARC domains-containing disease resistance protein**
AT5G63010	*Transducin/WD40 repeat-like superfamily protein*
**AT5G63020**	**Disease resistance protein (CC-NBS-LRR class) family**
